# Hypothalamic SIRT1 prevents age-associated weight gain by improving leptin sensitivity in mice

**DOI:** 10.1007/s00125-013-3140-5

**Published:** 2013-12-29

**Authors:** Tsutomu Sasaki, Osamu Kikuchi, Mayumi Shimpuku, Vina Yanti Susanti, Hiromi Yokota-Hashimoto, Ryo Taguchi, Nobuyuki Shibusawa, Takashi Sato, Lijun Tang, Kosuke Amano, Tomoya Kitazumi, Mitsutaka Kuroko, Yuki Fujita, Jun Maruyama, Yong-soo Lee, Masaki Kobayashi, Takashi Nakagawa, Yasuhiko Minokoshi, Akihiro Harada, Masanobu Yamada, Tadahiro Kitamura

**Affiliations:** 1 Metabolic Signal Research Center, Institute for Molecular and Cellular Regulation, Gunma University, 3-39-15 Showa-machi, Maebashi-shi, Gunma 371-8512 Japan; 2 Department of Medicine and Molecular Science, Graduate School of Medicine, Gunma University, Maebashi, Gunma Japan; 3 Laboratory of Molecular Traffic, Department of Molecular and Cellular Biology, Institute for Molecular and Cellular Regulation, Gunma University, Maebashi, Gunma Japan; 4 Division of Endocrinology and Metabolism, Department of Developmental Physiology, National Institute for Physiological Sciences, Okazaki, Aichi Japan; 5 Frontier Research Core for Life Science, University of Toyama, Toyama, Toyama Japan; 6 Department of Cell Biology, Graduate School of Medicine, Osaka University, Suita, Osaka Japan

**Keywords:** AgRP, Brown adipose tissue, Diet-induced obesity, Energy expenditure, Food intake, Hypothalamus, Leptin sensitivity, NAD^+^, POMC, Sympathetic activity

## Abstract

**Aims/hypothesis:**

Obesity is associated with ageing and increased energy intake, while restriction of energy intake improves health and longevity in multiple organisms; the NAD^+^-dependent deacetylase sirtuin 1 (SIRT1) is implicated in this process. Pro-opiomelanocortin (POMC) and agouti-related peptide (AgRP) neurons in the arcuate nucleus (ARC) of the hypothalamus are critical for energy balance regulation, and the level of SIRT1 protein decreases with age in the ARC. In the current study we tested whether conditional *Sirt1* overexpression in mouse POMC or AgRP neurons prevents age-associated weight gain and diet-induced obesity.

**Methods:**

We targeted *Sirt1* cDNA sequence into the *Rosa26* locus and generated conditional *Sirt1* knock-in mice. These mice were crossed with mice harbouring either *Pomc-Cre* or *Agrp-Cre* and the metabolic variables, food intake, energy expenditure and sympathetic activity in adipose tissue of the resultant mice were analysed. We also used a hypothalamic cell line to investigate the molecular mechanism by which *Sirt1* overexpression modulates leptin signalling.

**Results:**

Conditional *Sirt1* overexpression in mouse POMC or AgRP neurons prevented age-associated weight gain; overexpression in POMC neurons stimulated energy expenditure via increased sympathetic activity in adipose tissue, whereas overexpression in AgRP neurons suppressed food intake. SIRT1 improved leptin sensitivity in hypothalamic neurons in vitro and in vivo by downregulating protein-tyrosine phosphatase 1B, T cell protein-tyrosine phosphatase and suppressor of cytokine signalling 3. However, these phenotypes were absent in mice consuming a high-fat, high-sucrose diet due to decreases in ARC SIRT1 protein and hypothalamic NAD^+^ levels.

**Conclusions/interpretation:**

ARC SIRT1 is a negative regulator of energy balance, and decline in ARC SIRT1 function contributes to disruption of energy homeostasis by ageing and diet-induced obesity.

**Electronic supplementary material:**

The online version of this article (doi:10.1007/s00125-013-3140-5) contains peer-reviewed but unedited supplementary material, which is available to authorised users.

## Introduction

Obesity has been increasing worldwide over the past 30 years and has emerged as a global healthcare challenge [1]. Although a sedentary lifestyle is a major contributor to weight gain, during this time period the average energy intake has increased in many parts of the world [2]. Meanwhile, the global emergence of overweight and obesity is confounded by the simultaneous ageing of the population; the prevalence of obesity is positively associated with age [3, 4]. Conversely, caloric restriction improves the longevity and health of multiple organisms, including monkeys [5, 6]. Therefore, energy balance (energy intake) and ageing negatively correlate with health.

Monogenic models of obesity, such as mutations in the leptin and leptin receptor genes, have been useful for elucidating the molecular mechanisms underlying the regulation of energy balance [7, 8], although the monogenic form of obesity accounts for only a small portion of human obesity and results in massive obesity [9]. In contrast, weight gain occurring over the course of life is gradual and mild, and emphasizes the importance of paying attention to subtle phenotypes for elucidating the mechanisms of ageing-based disruptions in energy homeostasis.

Sirtuin 1 (SIRT1) is an NAD^+^-dependent deacetylase that serves as an energy sensor [10]. SIRT1 is the mammalian orthologue of the SIR2 protein found in *Saccharomyces cerevisiae*, and is crucial for caloric restriction-induced longevity [11–13]. Interestingly, single nucleotide polymorphisms in *SIRT1* are associated with obesity [14–17]. Thus, increasing evidence suggests that SIRT1 is a key regulator of whole-body energy balance and also plays a role in human health.

The central melanocortin system is crucial for hypothalamic control of whole-body energy balance. Anorexigenic pro-opiomelanocortin (POMC)-positive neurons and orexigenic Agouti-related protein (AgRP)-positive neurons are key players in this system; these neurons are mainly located in the arctuate nucleus (ARC) of the hypothalamus [18]. Contrary to the situation in most tissues, SIRT1 protein in the hypothalamus decreases with fasting and increases with feeding [19]. Since SIRT1 level decreases with age specifically in the ARC [20] and ageing is associated with positive energy balance, SIRT1 function in the ARC may negatively regulate energy balance. However, the results of pharmacological interventions, *Sirt1* stereotaxic overexpression and *Sirt1* knock-down studies have been inconsistent, leading to controversy regarding the role of hypothalamic SIRT1 in regulating energy balance [19, 21–27].

To test whether conditional *Sirt1* overexpression in mouse POMC or AgRP neurons prevents age-associated weight gain and diet-induced obesity, we created conditional *Sirt1* knock-in (KI) mice based on the *Rosa26* system [28], crossed with *Pomc-Cre* mice [29] or *Agrp-Cre* mice [30], and analysed the effect of *Sirt1* overexpression in POMC neurons or AgRP neurons.

## Methods

### Generation of Rosa26^Sirt1^ mice and mating

The pR26-1 plasmid was used to insert a conditional *Sirt1*-wild-type (WT) or enzyme-dead *Sirt1*-H355Y expression cassette into the *Rosa26* locus. Targeted embryonic stem cell clones were injected into C57BL/6 blastocysts to generate chimeras that transmitted the *Sw* (*Rosa26* ^Sirt1-WT^) or *Sh* (*Rosa26* ^Sirt1-H355Y^) allele to their progeny. Parent *Rosa26* mice were maintained on a 129/J × C57BL/6J background (one back-cross to C57BL/6J), and *Pomc-Cre* or *Agrp-Cre* heterozygous mice (C57BL/6J background) were used as the mating partners. The mating yielded mice heterozygous for *Sw* or *Sh* with or without a single *Cre* transgene allele. Siblings were born at the expected Mendelian ratio.

### PCR-based genotyping and detection of the recombinant locus

For standard genotyping, genomic DNA was extracted from tail samples and analysed by *Rosa26* genotyping PCR. For PCR identification of the *Rosa26* ^Sirt1-WT^ (Sw) and *Rosa26* ^Sirt1-H355Y^ (Sh) alleles, PCR was performed with the Sirt1-1918F primer and either the M13F or M13R primers (electronic supplementary material [ESM] Table 1). See ESM Methods for further details.

### Animal studies

All animal care and experimental procedures were approved by the Institutional Animal Care and Experimentation Committee at Gunma University. Mice were housed in individual cages in a temperature-controlled facility with a 12 h light/dark cycle. Mice were allowed free access to water and were given a standard laboratory chow diet (CLEA Rodent diet CE-2; CLEA Japan, Tokyo, Japan) unless otherwise specified. For the diet-induced obesity experiments, the high-fat high-sucrose (HFHS) diet (F2HFHSD; Oriental Yeast, Suita, Japan) consisted of protein (17.2% of energy intake), fat (54.5%) and carbohydrate (28.3%) and was fed to mice after weaning at 3 weeks of age. A multifeeder (MF-1M; Shinfactory, Fukuoka, Japan) was used for measuring HFHS diet intake. C57Bl/6, *db*/+ and *db*/*db* male mice were purchased from CLEA Japan.

### Indirect calorimetry and locomotor activity measurement

Oxygen consumption and CO_2_ production were measured in individual mice at the indicated age using an Oxymax apparatus (Columbus Instruments, Columbus, OH, USA). The O_2_ and CO_2_ measurements were performed every 18 min for each mouse over a 3 day period and the data from the final day were analysed. Locomotor activity was measured with the ACTIMO-100 (Shinfactory).

### Histological studies

Immunohistochemistry for hypothalamus and adipose tissue was performed using a standard protocol [19]. See ESM Methods for further details.

### Quantitative RT-PCR and western blot analysis

RNA extraction, cDNA synthesis, real-time PCR, cell lysate preparation and western analyses were performed using standard protocols [19]. Primer sequences are listed in ESM Table 2. A list of the antibodies used is shown in ESM Table 3. See ESM Methods for further details.

### Measurement of noradrenaline turnover

Noradrenaline (norepinephrine) turnover was measured on the basis of the decline in tissue noradrenaline content after the inhibition of catecholamine biosynthesis with α-methyl-*p*-tyrosine [31]. See ESM Methods for further details.

### Plasma and urine samples and rectal temperature

Measurement of these variables was performed with ELISA-based kits or by a thermistor. See ESM Methods for further details.

### T_3_ suppression test

Twelve-week-old male mice received daily i.p. injections of T_3_ (1.0 μg/100 g between 09:00 and 10:00 hours) for 6 days for a T_3_ suppression test. See ESM Methods for further details.

### Detection of acetylation in mouse embryonic fibroblast and brain lysates

Mouse embryonic fibroblasts (MEFs) were prepared using a standard protocol [32]. See ESM Methods for further details.

### Leptin studies in vivo and in vitro

Mice received i.p. injections of 0.5 μg/g leptin twice per day (at 8:00 and 17:30 hours) for 3 days and the effect was assessed. The effect of leptin was also assessed in hypothalamic N41 cells with or without electroporation of HA-tagged-ObRb (long isoform of leptin receptor). See ESM Methods for further details.

### Quantification of hypothalamic NAD^+^ levels by liquid chromatography–mass spectrometry

NAD^+^ was extracted from the hypothalamus with 0.5 mol/l perchloric acid, and the extract was centrifuged at 15,000 *g* for 10 min at 4°C. The supernatant fraction was neutralised with an equal volume of 1 mol/l ammonium formate. NAD^+^ levels were determined with an Agilent 6460 Triple Quad mass spectrometer coupled to an Agilent 1290 HPLC system (Agilent, Tokyo, Japan).

### Statistical analysis

Data are expressed as the mean value ± SEM. Statistical significance was assessed using Student’s *t* test. A *p* value of <0.05 was considered statistically significant.

## Results

### Characterisation of Rosa26^Sirt1-WT^ (Sw) mice and Rosa26^Sirt1-H355Y^ (Sh) mice

**Fig. 1:**
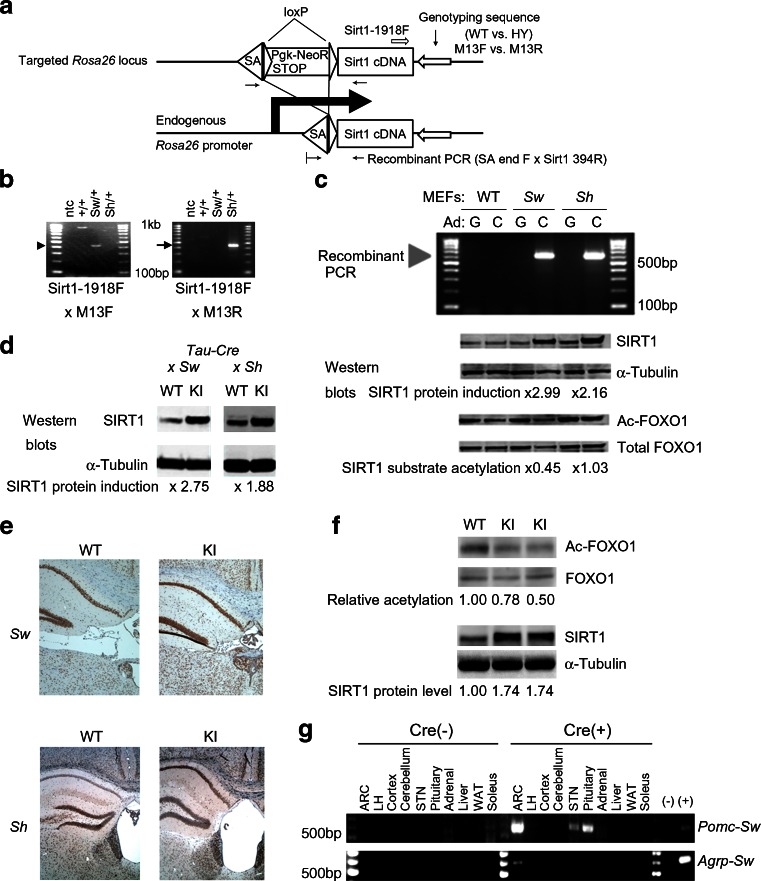
Generation of *Rosa26* ^*Sirt1*^ conditional KI mice. (**a**) Schematic figure of the targeted *Rosa26* locus and the position of the PCR primers used for genotyping. (**b**) PCR detection of the Sw locus and the Sh locus. The Sirt1-1918F and M13F primer set detects only the Sw locus (arrowhead), whereas the Sirt1-1918F and M13R primer set detects only the Sh locus (arrow). (**c**) Cre recombinase induces genetic recombination and *Sirt1* expression in MEFs from *Rosa26* ^*Sirt1-WT*^ (*Sw*) and *Rosa26* ^*Sirt1-H355Y*^ (*Sh*) mice. Note that Cre expression leads to a decrease in the acetylation of FOXO1 only in *Sw* MEFs. SIRT1 protein induction is based on the ratio of densitometries of SIRT1 and α-tubulin, and SIRT1 substrate acetylation is based on the ratio of densitometries of Ac-FOXO1 and total FOXO1. (**d**, **e**) SIRT1 protein expression can be induced in vivo. *Sw* and *Sh* mice were crossed with neuron-specific *Tau-Cre* mice, and SIRT1 protein expression was analysed by western blotting (**d**) or by immunohistochemistry (**e**). For (**e**), the panels above are from *Tau-Cre* × *Sw* mice and below are from *Tau-Cre* × *Sh* mice (magnification ×40). (**f**) Overexpression of *Sirt1* in neurons by crossing with *Tau-Cre* mice leads to induction of SIRT1 and decreased acetylation of FOXO1 in the forebrain of mice 1 h after TSA i.c.v. injection. (**g**) Genetic recombination in tissues from *Pomc-Cre*; *Rosa26* ^*Sirt1*^ mice and *Agrp-Cre*; *Rosa26* ^*Sirt1*^ mice. C, Cre; G, green fluorescent protein; HY, H355Y, LH; lateral hypothalmus; ntc, negative template control; SA, splicing acceptor; STN, solitary tract nucleus; WAT, white adipose tissue

To construct genetic gain-of-function models of *Sirt1* in mice, we introduced conditional *Sirt1* alleles into the *Rosa26* locus. In this system, the expression of either the wild-type (WT) *Sirt1* (Sw) or the enzyme-dead *Sirt1*-H355Y mutant (Sh) [33] is induced by Cre recombinase (Fig. 1a). Additional genotyping sequences were inserted 3′ to the *Sirt1* cDNA sequence so that the Sw locus and Sh locus were distinguishable by PCR (Fig. 1b). Induction of *Sirt1* by Cre recombinase was confirmed in MEFs (Fig. 1c) and in mice by crossing them with neuron-specific *Tau-Cre* mice [34] (Fig. 1d, e). Cre recombinase expression led to two- to threefold increases in SIRT1 protein levels compared with endogenous levels both in vitro and in vivo (Fig. 1c, d). Importantly, acetylation of forkhead box O1 (FOXO1), a known SIRT1 substrate, decreased when Cre recombinase was expressed in Sw MEFs but not in Sh fibroblasts (Fig. 1c). Overexpression of *Sirt1*-H355Y mutant in hypothalamic N41 cells or in MEFs did not alter the acetylation level of FOXO1, indicating that the Sh locus functions as a negative transgenic control, not as dominant-negative (ESM Fig. 1a, b). Because FOXO1 deacetylation decreases its stability [35], Sirt1 overexpression decreased total FOXO1 protein in vitro. We also analysed the acetylation status of FOXO1 in the forebrain samples of *Tau-Cre*; *Rosa26* ^*Sirt1-WT*^ (Sw) mice that received intracerebroventricular (i.c.v.) injection of trichostatin A (TSA, a histone deacetylase inhibitor) into the right lateral ventricle. Acetylation of FOXO1 was also decreased in KI mice, indicating that overexpression of *Sirt1* in this mouse model leads to increased SIRT1 activity in vivo (Fig. 1f). TSA treatment was necessary to detect the effect of SIRT1 deacetylase activity on FOXO1 (ESM Fig. 1a, b). We also tried to analyse the acetylation status of peroxisome proliferator-activated receptor γ coactivator 1α and nuclear factor κB p65 (two known SIRT1 substrates) but they were below the detection limit.

After confirming induced *Sirt1* expression in Sw and Sh mice, we crossed the mice with *Pomc-Cre* mice [29] or *Agrp-Cre* mice [30]. PCR genotyping of the recombinant locus confirmed the specificity of recombination (Fig. 1g) and also confirmed genetic recombination in the ARC, the solitary tract nucleus and the pituitary of *Pomc-Cre*-crossed Sw mice and the ARC of *Agrp-Cre*-crossed Sw mice. These observations were consistent with previous reports that characterised these Cre mice [29, 30]. These results demonstrated that the conditional Sw and Sh alleles were functional and that expression of WT or mutant *Sirt1* was induced in POMC neurons or in AgRP neurons by Cre recombinase.

### Overexpression of Sirt1 in POMC neurons results in a lean phenotype due to stimulated energy expenditure

**Fig. 2:**
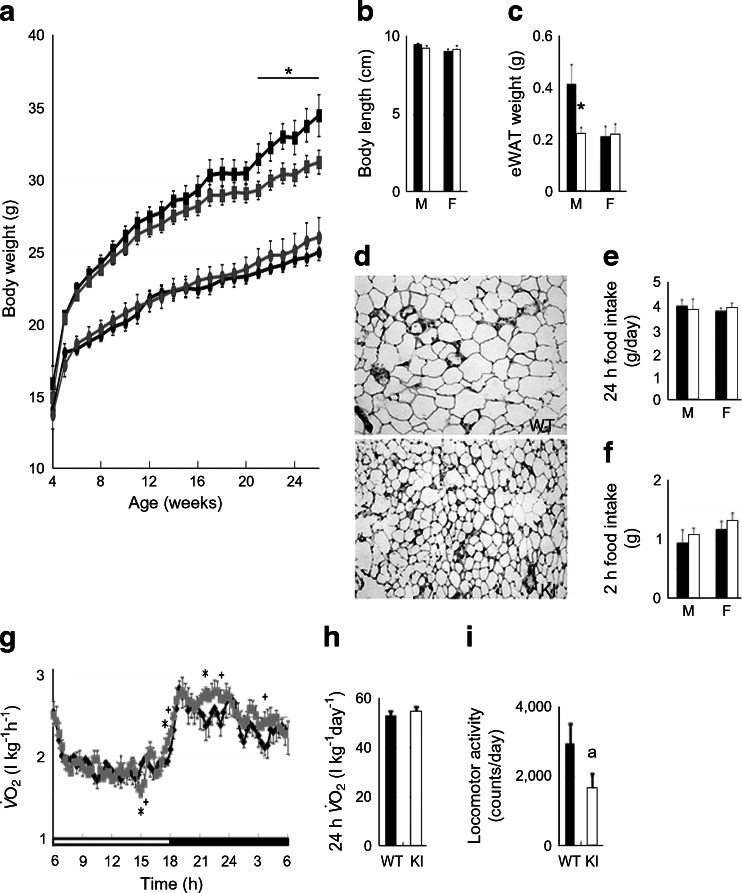
Overexpression of WT *Sirt1* in POMC neurons prevents age-associated weight gain in male mice. (**a**) Body weight curves of *Pomc-Cre*; *Rosa26* ^*Sirt1-WT*^ (*Sw*) conditional KI mice. (**b**, **c**) Body length (**b**) and epididymal white adipose tissue (eWAT) weight (**c**) of KI mice at 26 weeks of age. (**d**) Representative histological images of the eWAT of *Pomc-Cre*; *Rosa26* ^*Sirt1-WT*^ (*Sw*) mice at 26 weeks of age. (**e**, **f**) Twenty-four hour food intake (**e**) and 2 h food intake after 24 h fasting (**f**) of 25- to 26-week-old KI mice. (**g**–**i**) Oxygen consumption ($\overset{\cdot }{V}{\mathrm{O}}_2$) (**g**), 24 h $\overset{\cdot }{V}{\mathrm{O}}_2$ (**h**) and locomotor activity (**i**) of KI mice at 26 weeks of age. For *Sw* mice, WT males (black squares, *n* = 8), KI males (grey squares, *n* = 10), WT females (black circles, *n* = 10) and KI females (grey circles, *n* = 9) were analysed. Statistical analyses were performed using the two-tailed unpaired Student’s *t* test (^+^ *p* < 0.1 KI vs WT; *p < 0.05, KI vs WT). For (**i**), there was a trend (^a^ *p* = 0.08). Black bars, WT data; white bars, KI data. M, male mice; F, female mice

The body weights of *Pomc-Cre*; *Rosa26* ^*Sirt1*^ KI mice fed normal chow were monitored. After 20 weeks of age, male *Pomc-Cre*; *Rosa26* ^*Sirt1-WT*^ (Pomc-Sw) mice were protected from age-associated weight gain, whereas male *Pomc-Cre*; *Rosa26* ^*Sirt1-H355Y*^ (Pomc-Sh) mice were not (Fig. 2a and ESM Fig. 2a). The difference in body weight was not due to a difference in body length (Fig. 2b and ESM Fig. 2b); rather, it was due to decreased adiposity in the male Pomc-Sw mice (Fig. 2c, d and ESM Figs 2b and 3a, b).

We examined 24 h food intake and 2 h food intake after mice had been fasted for 24 h but the intakes did not differ from those of the controls (Fig. 2e, f and ESM Fig. 2d, e). Indirect calorimetry and analyses of locomotor activity revealed that male Pomc-Sw mice tend to have higher oxygen consumption (Fig. 2g, h and ESM Fig. 2f, g) and lower locomotor activity (Fig. 2i and ESM Fig. 2h). Since oxygen consumption captures basal metabolic rate, diet-induced thermogenesis and exercise, and because there was no difference in food intake, these data suggested that male Pomc-Sw mice experience increased energy expenditure due to higher basal metabolic rates. A similar case, in which elevated basal metabolic rate led to reduction of locomotor activity, has been reported [36].

### Pomc-Sw mice display enhanced sympathetic tone in adipose tissue and enhanced browning in subcutaneous white adipose tissue

**Fig. 3:**
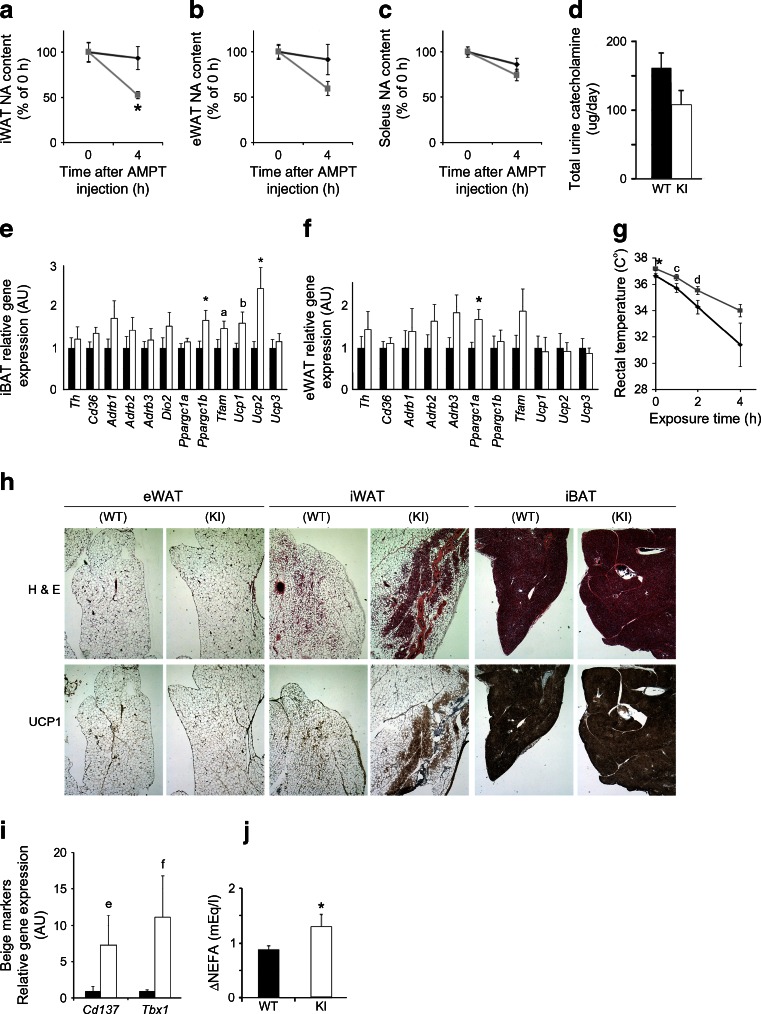
Increased sympathetic activity in the adipose tissue of *Pomc-Cre*; *Rosa26* ^*Sirt1-WT*^ (*Sw*) conditional KI mice. (**a**–**c**) Noradrenaline (NA) turnover in inguinal white adipose tissue (iWAT) (**a**), epididymal white adipose tissue (eWAT) (**b**) and the soleus (**c**) of 10-week-old male KI mice (*n* = 3 or 4). (**d**) Twenty-four hour urine catecholamine levels in 12-week-old male KI mice (WT, *n* = 7; KI, *n* = 6). (**e**, **f**) Gene expression profiles of interscapular brown adipose tissue (iBAT) (**e**) and eWAT (**f**) of 26-week-old male KI mice (WT, *n* = 8; KI, *n* = 10). (**g**) Rectal temperature of 16-week-old male KI mice during 4 h of cold exposure (4°C; WT, *n* = 10; KI, *n* = 7). (**h**, **i**) Enhanced browning of iWAT in KI mice. Representative haematoxylin and eosin (H&E) staining and UCP1 immunostaining, and gene expression profiles (**i**) of eWAT, iWAT and iBAT after 3 days of cold exposure (12-week-old mice; WT, *n* = 6; KI, *n* = 4). (**j**) Increased lipolysis in KI mice 15 min after stimulation with a β3 agonist (1 mg/kg of CL316243 i.p.) (WT, *n* = 7; KI, *n* = 4). Statistical analyses were performed using the two-tailed unpaired Student’s *t* test (*p < 0.05 KI vs WT). There were trends in (**e**) (^a^ *p* = 0.097; ^b^ *p* = 0.07), (**g**) (^c^ *p* = 0.097; ^d^ *p* = 0.067) and (**i**) (^e^ *p* = 0.09; ^f^ *p* = 0.05). Black bars and black lines, WT data; white bars and grey lines, KI data. AMPT, α-methyl-*p*-tyrosine

The central melanocortin system has been reported to regulate sympathetic activity in adipose tissues [37, 38]. Therefore, we measured noradrenaline turnover in tissues (a marker for sympathetic tone [31]) and 24 h urine catecholamine levels (a marker for systemic sympathetic tone) in male Pomc-Sw mice. Noradrenaline turnover was significantly increased in the inguinal white adipose tissue of KI mice and tended to increase in the epididymal white adipose tissue of KI mice (Fig. 3a, b). Increased sympathetic activity was not a systemic phenomenon, as neither the 24 h urine catecholamine levels nor the expression of β-adrenergic receptors and noradrenaline turnover in skeletal muscle were increased (Fig. 3c, d and ESM Fig. 4a).

The analysis of gene expression associated with sympathetic activity and mitochondrial function in brown adipose tissue and white adipose tissue revealed significantly increased mitochondrial activity (Fig. 3e, f). As a result, these mice tended to be cold-resistant for short-term exposures (Fig. 3g). Sympathetic activity regulates the browning of subcutaneous white adipose tissue induced by prolonged exposure to cold [39]. Therefore, we assessed adipose tissues after 3 days of exposure to cold. KI mice showed increased induction of uncoupling protein 1 (UCP1) and trends toward increased induction of the beige adipocyte markers *Cd137* (also known as *Tnfrsf9*) and *Tbx1* [40], indicating enhanced browning of subcutaneous white adipose tissue in these mice (Fig. 3h, i). Because sympathetic activity in white adipose tissue stimulates lipolysis, we compared plasma NEFA levels before and after stimulation with the β3 adrenergic agonist CL316243 [41] and found that the responses were enhanced in KI mice (Fig. 3j). In summary, male Pomc-Sw mice exhibited enhanced sympathetic activity in their adipose tissues.

Since genetic recombination was detected in the pituitaries of Pomc-Sw mice (Fig. 1g), we measured plasma corticosterone levels and ruled out an effect on the hypothalamus–pituitary–adrenal axis as a cause for the lean phenotype (ESM Fig. 4b). The hypothalamic melanocortin system can also regulate the hypothalamus–pituitary–thyroid axis [42], and male Pomc-Sw mice exhibited a trend toward increased plasma T_4_ levels accompanied by normal plasma levels of thyroid-stimulating hormone (TSH) (ESM Fig. 4c, d), suggesting a syndrome of inappropriate secretion of TSH. However, there were no significant differences in the degree of suppression of plasma TSH and pituitary *Tshb* expression during T_3_ suppression tests (ESM Fig. 4 e, f). Therefore, we conclude that the observed increased basal metabolic rate in male Pomc-Sw mice is mainly driven by increased sympathetic activity in adipose tissue and not by alterations in the hypothalamus–pituitary–adrenal or hypothalamus–pituitary–thyroid axes.

### Sirt1 overexpression in AgRP neurons controls body weight by regulating food intake

**Fig. 4:**
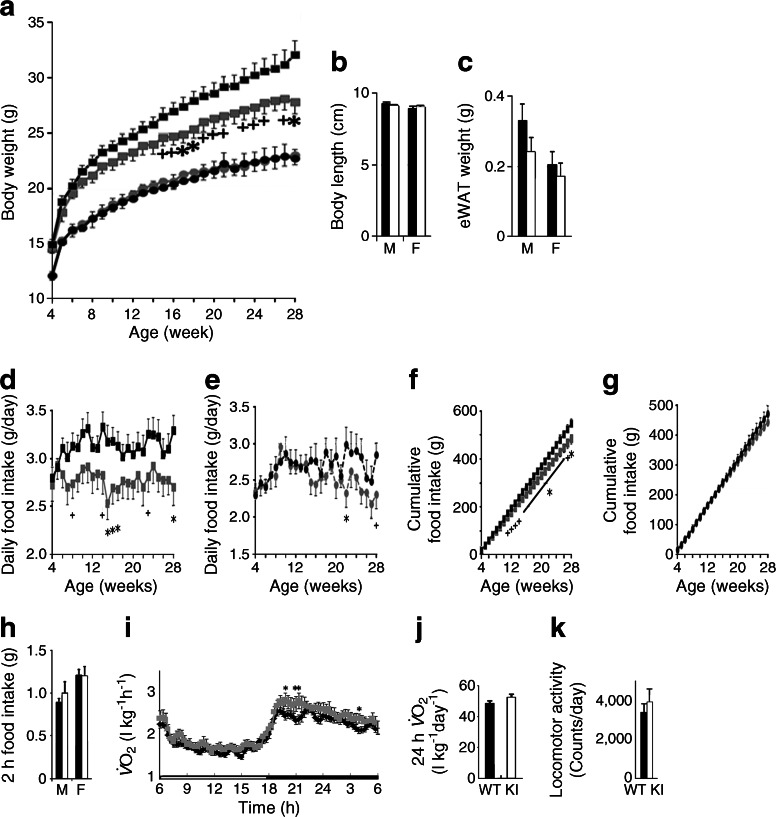
Overexpression of WT *Sirt1* in AgRP neurons prevents age-associated weight gain in male mice. (**a**) Body weight curves of *Agrp-Cre*; *Rosa26* ^*Sirt1-WT*^ (*Sw*) (black squares, WT males, *n* = 18; grey squares, KI males, *n* = 12; black circles, WT females, *n* = 9; grey circles, KI females, *n* = 8) conditional KI mice. (**b**, **c**) Body length (**b**) and epididymal white adipose tissue (eWAT) weight (**c**) of KI mice at 26 weeks of age. (**d**–**h**) Daily food intake of male (**d**) and female (**e**) mice, cumulative food intake of male (**f**) and female (**g**) mice and 2 h food intake (**h**) of KI mice after 24 h of fasting. (**i**–**k**) Oxygen consumption ($\overset{\cdot }{V}{\mathrm{O}}_2$) (**i**), 24 h $\overset{\cdot }{V}{\mathrm{O}}_2$ (**j**) and locomotor activity (**k**) of KI mice at 28 weeks of age. Black and grey symbols for all figure parts are as described for (**a**). Statistical analyses were performed using the two-tailed unpaired Student’s *t* test (^+^ *p* < 0.1; *p < 0.05 KI vs WT). Black bars, WT data; white bars, KI data. M, male; F, female

Next, we analysed the effect of *Sirt1* overexpression in AgRP neurons by crossing *Agrp-Cre* mice with Sw or Sh mice and feeding them with a normal chow diet. Male *Agrp-Cre*; *Rosa26* ^*Sirt1-WT*^ mice (Agrp-Sw) showed a lean phenotype, whereas female *Agrp-Cre*; *Rosa26* ^*Sirt1-H355Y*^ mice (Agrp-Sh) tended toward increased body weight (Fig. 4a and ESM Fig. 5a). There were no differences in the body length or adiposity of either Agrp-Sw or Agrp-Sh mice compared with control mice (Fig. 4b, c and ESM Fig. 5b, c). Decreased and increased food intake led to the observed differences in body weight of the Agrp-Sw male and Agrp-Sh female mice, respectively (Fig. 4d, h and ESM Fig. 5d, h). Even adjusted for their lighter body weight, Agrp-Sw male mice tended to eat less than their littermates (ESM Fig. 6a–d). In contrast to the Pomc-Sw mice, these mice did not exhibit any significant differences in energy expenditure, locomotor activity or mitochondrial gene expression in brown adipose tissue or in white adipose tissue compared with controls (Fig. 4i–k and ESM Figs 5i–k and 6e, f). Further, no differences in plasma T_4_ levels, fasting-induced lipolysis or rectal temperature were detected (ESM Figs 5l and 6g–i). Taken together, these data indicate that overexpression of *Sirt1* in AgRP neurons controls body weight by regulating food intake.

### SIRT1 improves leptin sensitivity in hypothalamic neurons both in vivo and in vitro

**Fig. 5:**
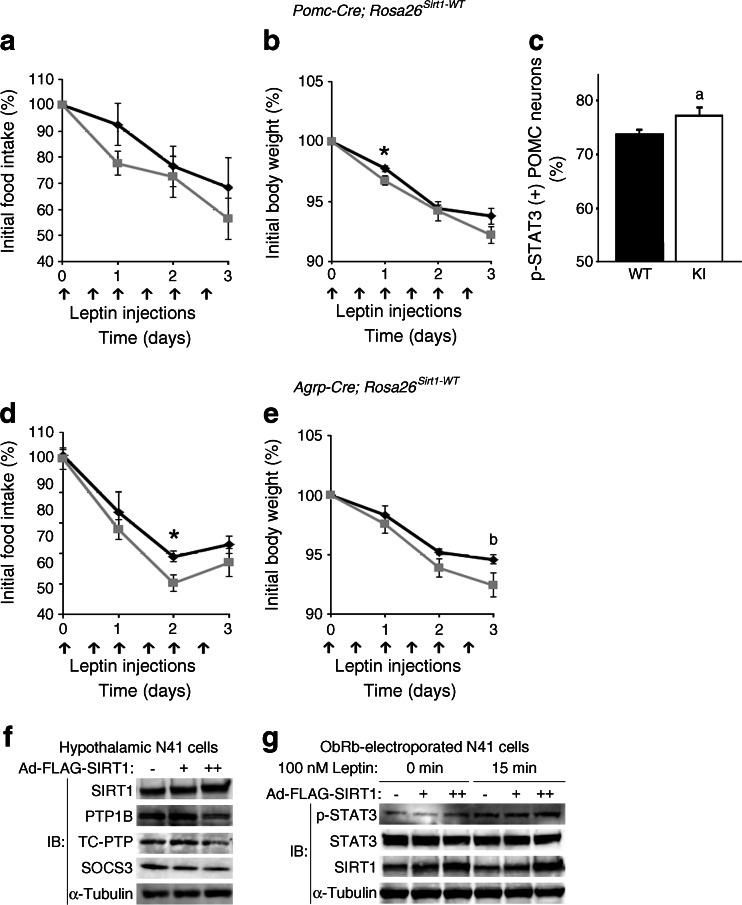
SIRT1 improves leptin sensitivity in hypothalamic neurons both in vivo and in vitro. (**a**–**c**) *Pomc*-*Sw* KI mice tended to be more sensitive to leptin. The effect of i.p. leptin (0.5 μg/g, twice per day) on food intake (**a**) and body weight (**b**) (WT, *n* = 7; KI, *n* = 6) and on POMC neuron activation 45 min after i.p. leptin (1 μg/μl) as measured by p-STAT3-positive POMC neurons in the ARC (**c**) (WT, *n* = 4; KI, *n* = 5) of 11- to 12-week-old male *Pomc*-*Sw* KI mice. (**d**, **e**) The effect of i.p. leptin injection on food intake (**d**) and body weight (**e**) of 11- to 12-week-old male *Agrp-Cre*; *Rosa26* ^*Sirt1-WT*^ mice (WT, *n* = 5; KI, *n* = 5). (**f**) *Sirt1* overexpression in hypothalamic N41 cells decreased levels of PTP1B, TC-PTP and SOCS3 protein. (**g**) *Sirt1* overexpression in ObRb-electroporated N41 cells increased phosphorylation of STAT3 upon leptin treatment. IB, Immunoblot. Statistical analyses were performed using the two-tailed unpaired Student’s *t* test (*p < 0.05). There were trends in (**c**) (^a^ *p* = 0.08) and (**e**) (^b^ *p* = 0.08). Black bars and black lines, WT data; white bars and grey lines, KI data

We next assessed the leptin sensitivity in our mice. Injection of a low dose of leptin intraperitoneally in 11- to 12-week-old male Pomc-Sw mice (which do not show the lean phenotype at this early age) demonstrated that the male KI mice tended to be leptin-sensitive (Fig. 5a, b) and tended to harbour more POMC neurons in the ARC that were positive for phosphorylated signal transducer and activator of transcription 3 (STAT3) (Fig. 5c). It suggests that this increased leptin sensitivity was the cause, not the consequence, of the lean phenotype. The KI mice showed decreased expression of *Agrp* and a trend for decreased *Npy* expression and increased activation of POMC neurons after 24 h of fasting as measured by c-fos activity, indicating increased sensitivity of POMC neurons to nutritional signals in the KI mice (ESM Fig. 7a–c). The number of POMC neurons or projections of POMC or AgRP fibres to the paraventricular nucleus did not differ from controls (ESM Fig. 7d, e). These data indicate that higher sensitivity to leptin and nutritional signals from the POMC neurons of Pomc-Sw mice led to increased sympathetic activity in adipose tissues, resulting in increased energy expenditure in Pomc-Sw male mice.

The leptin sensitivity was also enhanced in young Agrp-Sw mice (Fig. 5d, e). The expression of *Pomc*, *Agrp* and *Npy*, projections of POMC neurons and AgRP neurons to the paraventricular nucleus, and the number of AgRP neurons did not differ between WT and KI mice (ESM Fig. 7f–i). Therefore, overexpression of *Sirt1* in AgRP neurons increases leptin sensitivity, which is known to suppress the activity of AgRP neurons, and suppressed food intake. In summary, *Sirt1* overexpression in the ARC improves leptin sensitivity in mice.

Next, the expression levels of *Sirt1* were manipulated in a hypothalamic N41 cell line via adenovirus encoding *Sirt1*. Overexpression of *Sirt1* decreased the protein levels of protein-tyrosine phosphatase 1B (PTP1B), T cell protein-tyrosine phosphatase (TC-PTP) and suppressor of cytokine signalling 3 (SOCS3), which are known to contribute to leptin resistance [43] (Fig. 5f, g). As a consequence, phosphorylation of Tyr705 of STAT3 was enhanced by *Sirt1* overexpression in ObRb-expressing N41 cells (Fig. 5g). Therefore, overexpression of *Sirt1* improves leptin signalling in hypothalamus cells in vitro by reducing levels of PTP1B, TC-PTP and SOCS3.

### An HFHS diet suppresses the lean phenotype due to Sirt1 overexpression in POMC neurons or AgRP neurons

**Fig. 6:**
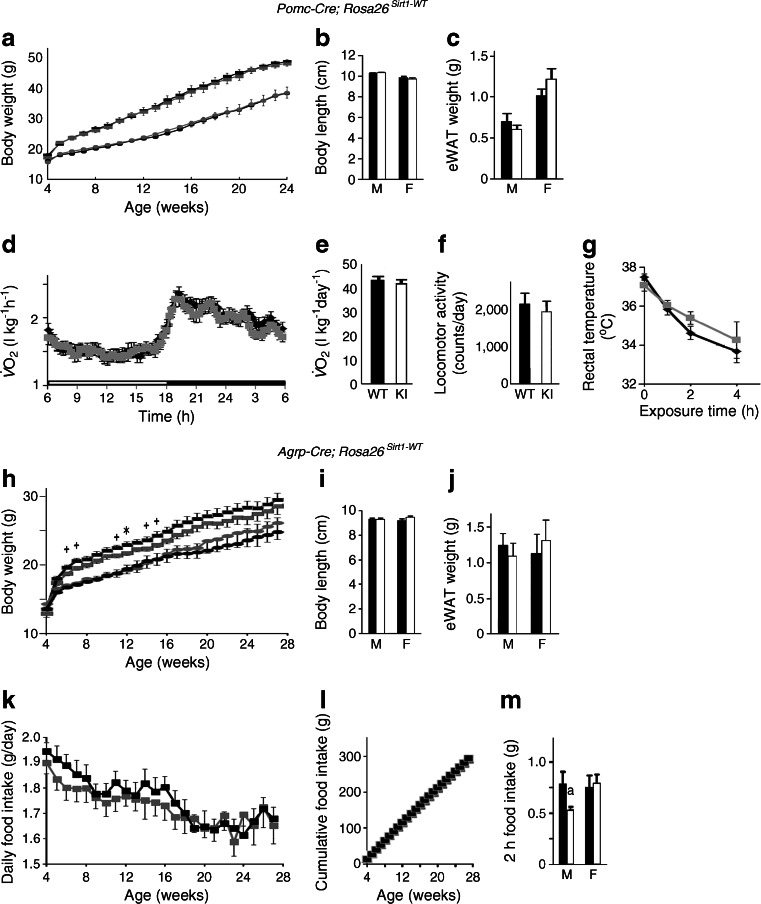
*Sirt1* overexpression in POMC neurons or in AgRP neurons does not prevent diet-induced obesity. (**a**) Body weight curves of *Pomc-Cre*; *Rosa26* ^*Sirt1-WT*^ (*Sw*) conditional KI mice fed an HFHS diet starting at 3 weeks of age (black squares, WT males, *n* = 14; grey squares, KI males, *n* = 12; black circles, WT females, *n* = 12; grey circles, KI females, *n* = 13). (**b**, **c**) The body length (**b**) and epididymal white adipose tissue (eWAT) weight (**c**) of 26-week-old KI mice fed an HFHS diet. (**d**–**f**) Oxygen consumption ($\overset{\cdot }{V}{\mathrm{O}}_2$) (**d**), 24 h $\overset{\cdot }{V}{\mathrm{O}}_2$ (**e**) and locomotor activity (**f**) of 26-week-old male KI mice fed an HSHS diet. (**g**) Rectal temperature of 26-week-old male KI mice fed an HSHS diet during exposure to cold (4°C) for 4 h (WT, *n* = 8; KI, *n* = 7). (**h**) Body weight of *Agrp-Cre*; *Rosa26* ^*Sirt1-WT*^ (*Sw*) conditional KI mice fed an HFHS diet starting at 3 weeks of age (black squares,WT male, *n* = 13; grey squares, KI male, *n* = 14; black circles, WT female, *n* = 9; grey circles, KI female, *n* = 13). (**i**, **j**) Body length (**i**) and eWAT weight (**j**) of 28-week-old KI mice fed an HFHS diet. (**k**–**m**) Daily food intake (**k**), cumulative food intake (**l**) and 2 h food intake after 24 h fasting (**m**) of male KI mice fed an HFHS diet. Statistical analyses were performed using two-tailed unpaired Student’s *t* tests (^+^ *p* < 0.1; *p < 0.05 KI vs WT). There was a trend in (**m**) (^a^ *p* = 0.08). Black bars and black lines, WT data; white bars and grey lines, KI data. M, male; F, female

We next tested whether *Sirt1* overexpression in POMC neurons or AgRP neurons protects mice from diet-induced obesity by feeding them an HFHS diet. Contrary to our expectations, these mice were not protected from diet-induced obesity. The Pomc-Sw mice gained weight at a rate similar to that shown by their WT littermates, with mean body length remaining unchanged (Fig. 6a, b). Mice gained more epididymal white adipose tissue weight than normal chow-fed mice, but there was no difference between WT and KI mice (Figs 2c and 6c). Loss of the lean phenotype appears to be due to a loss of increased energy expenditure. Energy expenditure and locomotor activity did not differ from controls, nor did food intake (Fig. 6d–f and ESM Fig. 8a, b). As a consequence, these mice also lost resistance to cold (Fig. 6g). Meanwhile, male Agrp-Sw mice were leaner than their WT littermates at an early age. However, the lean phenotype was lost over time when the mice received an HFHS diet, and body length remained unchanged (Fig. 6h, i). HFHS diet-fed WT and KI mice both gained more epididymal white adipose tissue weight than normal chow-fed mice, and there was no difference between HFHS diet-fed WT and KI mice (Figs 4c and 6j). Energy expenditure, locomotor activity and cold tolerance in these mice did not differ from controls (ESM Fig. 8c–f). Loss of the lean phenotype coincided with loss of the trend towards reduced HFHS diet intake over time (Fig. 6k–m). Thus, an HFHS diet suppresses the lean phenotype caused by *Sirt1* overexpression both in POMC neurons and in AgRP neurons in a dose-dependent manner.

### An HFHS diet decreases ARC SIRT1 activity by decreasing hypothalamic NAD^+^ content and ARC SIRT1 protein levels

**Fig. 7:**
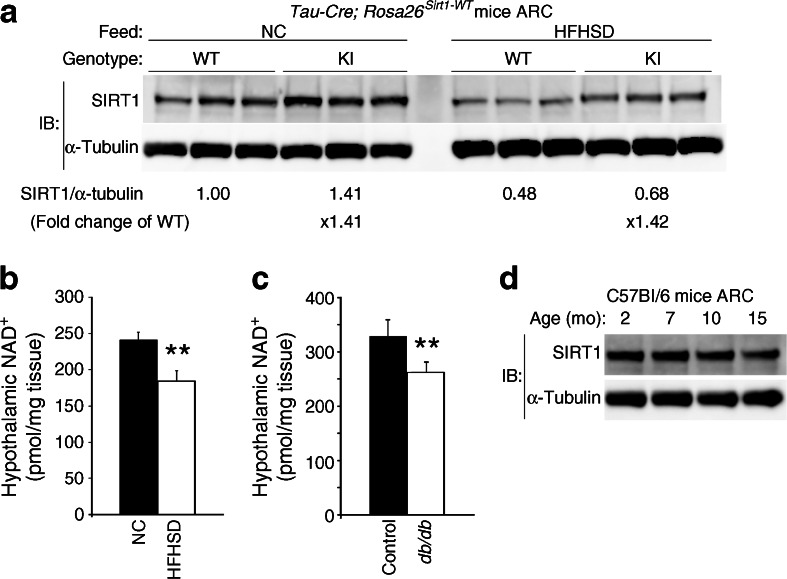
HFHS diet suppresses the function of ARC SIRT1. (**a**) Western blot of ARC samples taken from male *Tau-Cre*; *Rosa26* ^*Sirt1-WT*^ mice at 8 week of age. These mice were fed either normal chow (NC) or an HFHS diet (HFHSD) for 4 weeks. Each lane was loaded with 10 μg of sample from a single mouse ARC. (**b**, **c**) Hypothalamic NAD^+^ levels in 7-week-old C57Bl/6 male mice fed either NC (black bars, *n* = 6) or an HFHSD (white bars, *n* = 7) for 4 weeks (**b**) and in male control mice (black bars, *n* = 5) and male *db*/*db* mice (white bars, *n* = 7) at 8 month of age (**c**). (**d**) Western blot of ARC samples from male C57Bl/6 mice at various ages. IB, immunoblot. Statistical analyses were performed using two-tailed unpaired Student’s *t* tests (**p < 0.01)

Next, we tested whether the HFHS diet decreased levels of SIRT1 protein and/or NAD^+^ (a co-factor required for the enzymatic activity of SIRT1) in the ARC. To assess the effect of the HFHS diet on overexpressed SIRT1 protein levels in the ARC, we overexpressed *Sirt1* in all neurons by using male neuron-specific *Tau-Cre*; *Rosa26* ^*Sirt1-WT*^ mice (neuronal-Sw) and analysed their ARC. SIRT1 protein level in the ARC was attenuated following 4 weeks of consumption of the HFHS diet compared with consumption of normal chow (Fig. 7a).

We also analysed the impact of diet-induced obesity and genetically induced obesity on hypothalamic NAD^+^ content by comparing male B6 mice fed either normal chow or the HFHS diet for 4 weeks after weaning, and by comparing 8-month-old male *db*/*db* mice and their littermate controls. Hypothalamic NAD^+^ content was significantly decreased in both HFHS diet-fed mice and in *db*/*db* mice compared with their control groups (Fig. 7b, c). Therefore, the HFHS diet suppressed the effect of *Sirt1* overexpression in the ARC by decreasing the levels of SIRT1 protein overexpression and hypothalamic NAD^+^.

## Discussion

**Fig. 8:**
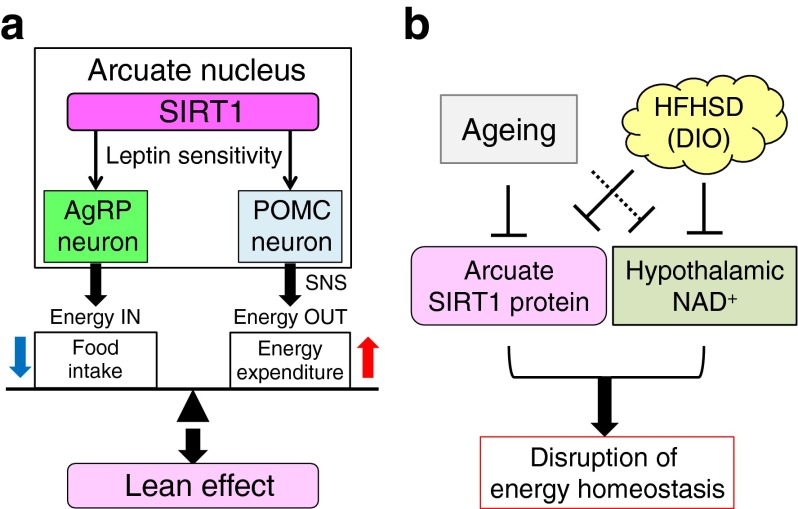
A proposed model for the action of ARC SIRT1 in negatively regulating energy balance, and the effect of ageing and obesity on this machinery. (**a**) ARC SIRT1 negatively regulates energy balance through improved leptin sensitivity in AgRP neurons and POMC neurons. (**b**) Both ageing and obesity suppress ARC SIRT1 and hypothalamic NAD^+^ levels, which disrupt the energy homeostasis regulated by ARC SIRT1. DIO, diet-induced obesity; SNS, sympathetic nervous system

In this study, we generated conditional *Sirt1* KI mouse models (Fig. 1a–g) and asked whether conditional *Sirt1* overexpression in mouse POMC or AgRP neurons prevents age-associated weight gain and diet-induced obesity. We found that *Sirt1* overexpression in hypothalamic POMC or AgRP neurons prevents age-associated weight gain. SIRT1 in POMC neurons stimulated energy expenditure through enhancing sympathetic activity in adipose tissue (Figs 2a–i and 3a–j), and SIRT1 in AgRP neurons suppressed food intake (Fig. 4a–k). *Sirt1* overexpression suppressed PTP1B, TC-PTP and SOCS3 and improved leptin sensitivity (Fig. 5a–g). However, *Sirt1* overexpression in these neurons did not protect mice from diet-induced obesity, because an HFHS diet negatively regulated ARC SIRT1 protein levels and hypothalamic NAD^+^ content (Figs 6a–m and 7a–c). Considering that ARC SIRT1 protein levels decrease with age (Fig. 7d) and that NAD availability in the hypothalamus is likely to decrease with age [44, 45], these findings define ARC SIRT1 as a negative regulator of energy balance and indicate that the decline in ARC SIRT1 function due to excessive energy intake or ageing contributes to age-associated weight gain (Fig. 8a, b).

Although there are mixed reports regarding the role of hypothalamic SIRT1 in energy balance regulation, results of *Sirt1* overexpression in POMC neurons were consistent with a previous report on genetic deletion of *Sirt1* in POMC neurons [25]. On the other hand, genetic deletion of *Sirt1* in AgRP neurons has been reported to result in reduced food intake in female mice fed a normal chow diet [26]. Therefore, our results appeared at first to be the opposite of the previous AgRP neuron-specific *Sirt1* knock-out report: either overexpressing *Sirt1* (our study) or deleting *Sirt1* [26] results in suppressed food intake. Mechanistically, however, overexpressing *Sirt1* in AgRP neurons increases leptin sensitivity, which is known to suppress the activity of AgRP neurons; the loss of *Sirt1* in AgRP neurons decreases the excitability of orexigenic AgRP neurons. Therefore, both genetic manipulations resulted in reduced food intake by suppressing AgRP neurons in different manners.

Our overall analyses indicate that leanness observed in mice overexpressing *Sirt1* in POMC neurons was associated with enhanced sympathetic activity in adipose tissue, leading to increased energy expenditure in these mice. However, it has been reported that dividing indirect calorimetry data by body weight might lead to spurious interpretations [46]. Thus, we cannot rule out the possibility that POMC-*Sirt1* KI mice may have had an energy expenditure that was appropriate for their reduced body weight.

We noted that the lean phenotype observed in Pomc-Sw mice was similar to that seen in mice in which *Lepr* is re-expressed only in POMC neurons in the ARC in a *Lepr*-KO background [47]. Mice in both genetic models undergo an increase in energy expenditure without an effect on food intake; these phenotypes have only been observed in males. *Lepr*-mediated regulation of energy balance via POMC neurons is known to be sexually dimorphic [48]. Therefore, male-specific phenotype observed by *Sirt1* overexpression could be due to modulation of leptin signalling. We do not believe that the sexually dimorphic phenotypes observed in our mice come from the effect on sex steroids because both male and female Pomc-Sw mice are fertile and the number of offspring is not different from WT littermates (data not shown). Another possible mechanism for the sexual dimorphism is that the dose requirement for SIRT1 may be different in males and females. Only female Agrp-*Sirt1* knockout mice showed decreased normal chow intake [26], and only female Agrp-*Sirt1*-H355Y mice showed increased food intake. So AgRP neurons in female mice may be more susceptible to the decreased SIRT1 function.

Another important finding in this work was that consumption of an HFHS diet decreases hypothalamic NAD^+^ content. The expression level of intracellular nicotinamide phosphoribosyltransferase, an essential enzyme in the NAD biosynthetic pathway producing nicotinamide mononucleotide, is very low in brain and pancreas [49]. Therefore, these organs rely on circulating nicotinamide mononucleotide for NAD biosynthesis. Supplementation with an NAD intermediate increases NAD^+^ levels in peripheral tissues such as liver and skeletal muscle, but not in brain [50]. Therefore, the detailed mechanisms regulating hypothalamic NAD^+^ levels during ageing and diet-induced obesity require further investigation.

Another potential mechanism for the loss of the lean phenotype following consumption of an HFHS diet by mice overexpressing *Sirt1* in the ARC is the reduction of SIRT1 in the ARC. We previously reported that the ubiquitin–proteasome system specifically decreases SIRT1 protein levels in the hypothalamus during fasting, and that this mechanism is disrupted by an HFHS diet [19]. We also found that ARC SIRT1 protein levels decrease with age (Fig. 7d). Clarifying the detailed mechanisms that regulate ARC SIRT1 protein in the context of ageing and diet-induced obesity is our next goal.

In conclusion, the present investigation demonstrated that countering the age-dependent reduction in ARC SIRT1 levels by overexpressing *Sirt1* in POMC neurons or AgRP neurons prevents age-associated weight gain by improving leptin sensitivity; an HFHS diet negates these benefits by suppressing ARC SIRT1 function. Elucidating the exact mechanism by which an HFHS diet and ageing decrease ARC SIRT1 protein and, possibly, NAD biosynthesis may motivate the development of new therapeutics to treat diet-induced obesity and to preserve energy homeostasis during ageing.

## Addendum

Overexpression of *Sirt1* in the dorsomedial hypothalamus and lateral hypothalamus was recently reported to extend lifespan and to delay ageing in mice [51].

## Electronic supplementary material

Below is the link to the electronic supplementary material.

ESM Methods (PDF 102 kb)

ESM Fig. 1 (PDF 76 kb)

ESM Fig. 2 (PDF 157 kb)

ESM Fig. 3 (PDF 115 kb)

ESM Fig. 4 (PDF 136 kb)

ESM Fig. 5 (PDF 123 kb)

ESM Fig. 6 (PDF 155 kb)

ESM Fig. 7 (PDF 147 kb)

ESM Fig. 8 (PDF 148 kb)

ESM Table 1 (PDF 26 kb)

ESM Table 2 (PDF 29 kb)

ESM Table 3 (PDF 42 kb)

## Abbreviations

AgRP: Agouti-related protein
ARC: Arcuate nucleus
FOXO1: Forkhead box O1
HFHS: High-fat high-sucrose
KI: Knock-in
MEFs: Mouse embryonic fibroblasts
POMC: Pro-opiomelanocortin
PTP1B: Protein-tyrosine phosphatase 1B
SIRT1: Sirtuin 1
SOCS3: Suppressor of cytokine signalling 3
STAT3: Signal transducer and activator of transcription 3
TC-PTP: T cell protein-tyrosine phosphatase
TSA: Trichostatin A
TSH: Thyroid-stimulating hormone
UCP1: Uncoupling protein 1
WT: Wild-type
