# Supplementary material for: Hypothalamic SIRT1 prevents age-associated weight gain by improving leptin sensitivity in mice
Source: Diabetologia. 2013 Dec 29;57(4):819–31. doi: 10.1007/s00125-013-3140-5 (PMC3940852; doi:10.1007/s00125-013-3140-5)
Supplement: Supplementary file 1 — (PDF 102 kb) [file 125_2013_3140_MOESM1_ESM.pdf]

ESM Methods

PCR-based genotyping and detection of the recombinant locus. For standard genotyping, genomic DNA extracted from tail samples and analysed by *Rosa26* genotyping PCR using ExTaq polymerase (TAKARA BIO, Otsu, Japan) as described previously [1]. For PCR identification of the *Rosa26*$^{Sirt1\text{-}WT}$ (Sw) and *Rosa26*$^{Sirt1\text{-}H355Y}$ (Sh) alleles, PCR was performed with the Sirt1-1918F primer and either the M13F or M13R primers (**Table S1**). For PCR detection of the recombinant locus in mouse embryonic fibroblasts or dissected tissue samples, DNA from the samples was amplified using PrimeSTAR HS polymerase with GC-rich buffer (TAKARA Bio) with the SA end F and Sirt1-394R primers (**Table S1**). The recombined locus was detected from tail genomic DNA in approximately 12% of *Agrp-Cre*-crossed mice, which were removed from the study (data not shown).

Histological studies. Hypothalamic immunohistochemistry was performed as described previously [2]. Adipose tissues were fixed in 4% paraformaldehyde and embedded in paraffin. Paraffin sections were stained with hematoxylin and eosin or with anti-UCP1 antibody. Adipocyte size was measured as described previously [3]. For quantification of POMC or AgRP neuron projection to the paraventricular nucleus of the hypothalamus, we used a method similar to that described by [4]. We analysed data from five paraventricular nucleus sections per animal for signal quantitation.

Quantitative RT-PCR and western blot analysis. RNA extraction, cDNA synthesis and cell lysates were prepared as described previously [2]. Real-time PCR was performed using the LightCycler system and LightCycler 480 SYBR Green I (Roche Diagnostics). Primer sequences are listed in **Table S2**. Protein signals were visualized with an LAS4000 imager (FUJIFILM, Tokyo, Japan) and the signals were quantified using Image-J software (NIH). List of antibodies used is available as **Table S3**.

Measurement of noradrenaline turnover. Norepinephrine (noradrenaline) turnover was measured on the basis of the decline in tissue noradrenaline content after the inhibition of catecholamine biosynthesis with α-methyl-p-tyrosine (AMPT, 200 mg/kg, i.p.; Sigma, Tokyo, Japan, M3281), as described previously [5]. At 0 and 4 h after AMPT injection, the animals were decapitated and tissues were rapidly removed and weighed, then frozen in liquid nitrogen. The frozen tissue samples were homogenized in 0.2 mol/l perchloric acid containing 0.1 mmol/l ethylenediaminetetraacetic acid, the homogenates

were centrifuged at 4 °C, and the norepinephrine content of the resulting supernatants was assayed by high-performance liquid chromatography (EP-300 system; Eicom, Kyoto, Japan) with a reversed-phase column (CA-5ODS, Eicom) and electrochemical detector (ECD-300, Eicom). Data are expressed as percentage of norepinephrine content (picograms of norepinephrine per milligram of tissue weight) prior to the AMPT injection in the corresponding genotype of mice.

Plasma and urine samples and rectal temperature. Plasma non-esterified fatty acid was measured using the LabAssay NEFA kit (Wako, Tokyo, Japan). Plasma cortisol levels were measured using the Corticosterone EIA Kit (Cosmo Bio, Tokyo, Japan). Plasma T4 levels were measured using the Rodent T4 ELISA kit (Endocrine Technologies, Newark, CA, USA). These plasma samples were obtained between 20:00 and 21:00. For 24-h urine catecholamine measurement, 12-week-old mice were acclimated to the metabolic chambers for 5 days, and then urine samples were collected over a 24-h period. Total urine catecholamine levels in the samples were measured by SRL (Tokyo, Japan). Rectal temperature was measured using the D717 pocket-sized thermistor (Takara Thermistor, Yokohama, Japan).

T3 suppression test. Twelve-week-old male mice received daily i.p. injections of T3 (1.0 μg/100 g between 09:00 and 10:00) for 6 days. Two hours after the last injection, mice were anesthetized with pentobarbital and their pituitary and plasma were harvested. Plasma T4 levels were measured by enzyme-linked immunosorbent assay. Plasma TSH levels were measured by radioimmunoassay, as described previously [6]. Pituitary *Tshb* levels were measured by quantitative reverse transcription PCR with TaqMan probes for *Tshb* (Mm00437190_ml) and *Gapdh* (Mm99999915_gl) (purchased from Life Technologies, Tokyo, Japan).

Detection of acetylation in mouse embryonic fibroblast and brain lysates. Mouse embryonic fibroblasts were prepared as previously described [7]. In order to detect the difference in the acetylation status of Sirt1 substrates in brain lysates of *Tau-Cre;* $Rosa26^{Sirt1\text{-}WT}$ mice, these mice received stereotaxic implantation of a guide cannula into lateral ventricle as previously described using the following stereotaxic coordinates: 0.20 mm posterior, 1.00 mm right, and 2.50 mm depth from Bregma [2]. Two days after the surgery, mice were anesthetized and received intracerebroventricular injection of 0.5 ul of trichostatin A (10 ug/ul) through the lateral ventricular cannula. Mice were sacrificed 1 hour later and their forebrain harvested for further analyses.

Leptin studies in vivo and in vitro. Mice received i.p. injections of 0.5 μg/g leptin twice per day (at 8:00 and 17:30) for 3 days. Food intake and body weight were measured daily. For the p-STAT3 studies, mice that had fasted for 24 h received i.p. injections of 1 μg/g leptin and were perfused 45 min later. For N41 cell experiments, N41 cells with or without electroporation of pCI-HA-ObRb [8] via the Nucleofector L or V kit (Lonza, Tokyo, Japan) were infected with adenovirus carrying the gene encoding Sirt1. After serum removal for 24 h, cells were stimulated with or without 100 nmol/l leptin and harvested 15 min later.

ESM references

[1] Soriano P (1999) Generalized lacZ expression with the ROSA26 Cre reporter strain. Nat Genet 21: 70-71

[2] Sasaki T, Kim HJ, Kobayashi M, et al. (2010) Induction of hypothalamic Sirt1 leads to cessation of feeding via agouti-related peptide. Endocrinology 151: 2556-2566

[3] Kim HJ, Kobayashi M, Sasaki T, et al. (2012) Overexpression of FoxO1 in the hypothalamus and pancreas causes obesity and glucose intolerance. Endocrinology 153: 659-671

[4] Banno R, Zimmer D, De Jonghe BC, et al. (2010) PTP1B and SHP2 in POMC neurons reciprocally regulate energy balance in mice. J Clin Invest 120: 720-734

[5] Saito M, Minokoshi Y, Shimazu T (1989) Accelerated norepinephrine turnover in peripheral tissues after ventromedial hypothalamic stimulation in rats. Brain Res 481: 298-303

[6] Nakahara M, Johnson K, Eckstein A, et al. (2012) Adoptive transfer of antithyrotropin receptor (TSHR) autoimmunity from TSHR knockout mice to athymic nude mice. Endocrinology 153: 2034-2042

[7] Sasaki T, Maier B, Bartke A, Scrable H (2006) Progressive loss of SIRT1 with cell cycle withdrawal. Aging Cell 5: 413-422

[8] Belouzard S, Delcroix D, Rouille Y (2004) Low levels of expression of leptin receptor at the cell surface result from constitutive endocytosis and intracellular retention in the biosynthetic pathway. J Biol Chem 279: 28499-28508
