# Supplementary material for: Hypothalamic SIRT1 prevents age-associated weight gain by improving leptin sensitivity in mice
Source: Diabetologia. 2013 Dec 29;57(4):819–31. doi: 10.1007/s00125-013-3140-5 (PMC3940852; doi:10.1007/s00125-013-3140-5)
Supplement: Supplementary file 2 — (PDF 76 kb) [file 125_2013_3140_MOESM2_ESM.pdf]

ESM Fig. 1

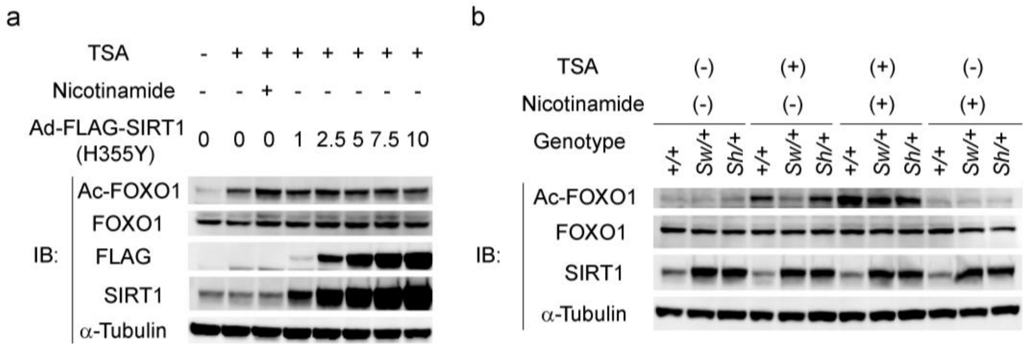

ESM Fig. 1, related to Fig. 1c. *Sirt1*-H355Y function as null-mutant, not dominant-negative *in vitro*. (**a**) Adenoviral over-expression of *Sirt1*-H355Y in hypothalamic N41 cells does not increase the acetylation of FOXO1. (**b**) *Sirt1*-H355Y expressed from the *Sh* locus does not increase the acetylation of FOXO1 in MEFs, whereas wild-type *Sirt1* expressed from the *Sw* locus promotes the deacetylation of FOXO1. Note that the presence of trichostatin-A is required to detect the effect of SIRT1 deacetylase activity on FOXO1.
