# Supplementary material for: Hypothalamic SIRT1 prevents age-associated weight gain by improving leptin sensitivity in mice
Source: Diabetologia. 2013 Dec 29;57(4):819–31. doi: 10.1007/s00125-013-3140-5 (PMC3940852; doi:10.1007/s00125-013-3140-5)
Supplement: Supplementary file 3 — (PDF 157 kb) [file 125_2013_3140_MOESM3_ESM.pdf]

ESM Fig. 2

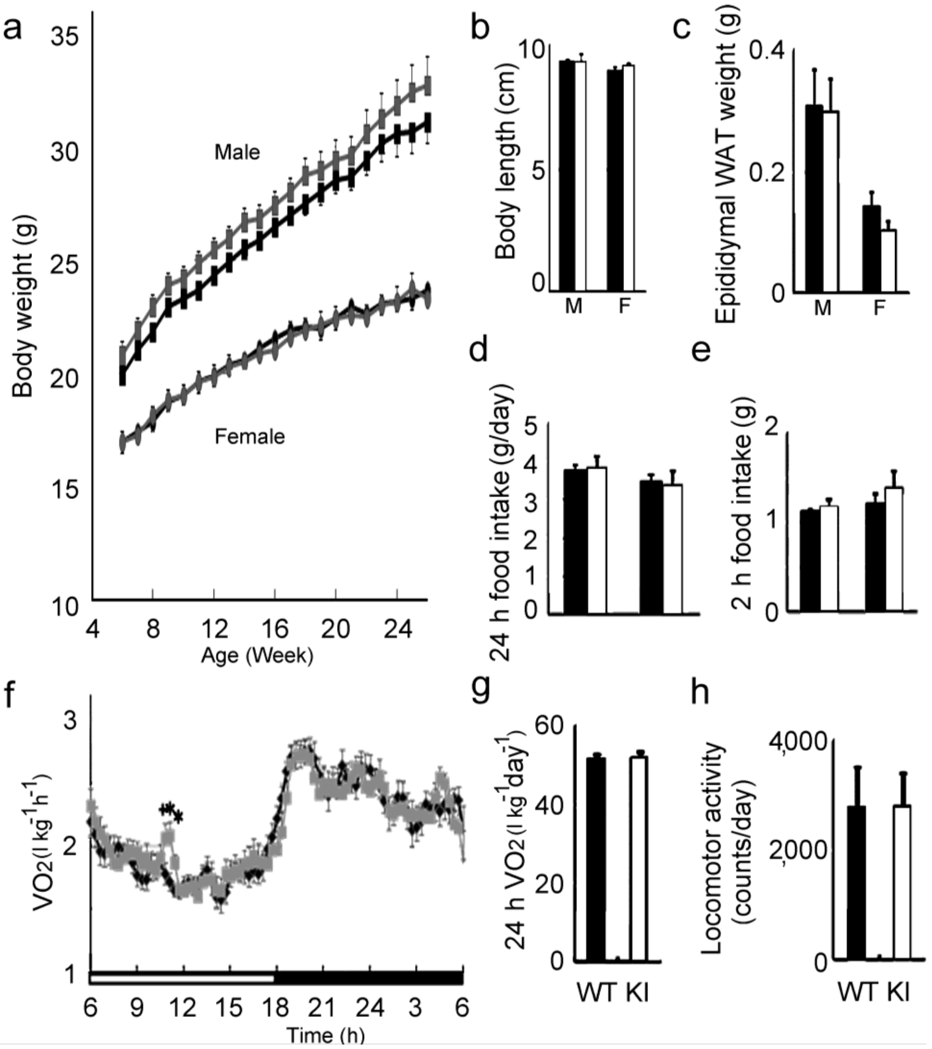

ESM Fig. 2, related to Fig. 2a-i. Phenotypes of *Pomc-Cre; Rosa26* $^{\mathit{Sirt1\text{-}H355Y}}$ mice. (**a**) Body-weight curves for *Pomc-Cre; Rosa26* $^{\mathit{Sirt1\text{-}H355Y}}$ (*Sh*) conditional KI mice. (**b, c**) Body length (**b**) and epididymal white adipose tissue (eWAT) weight (**c**) of KI mice at 26 weeks of age. (**d, e**) 24 h food intake and 2 h food intake after 24 h fasting (**e**) of 25- to 26-week-old KI mice. (**f-h**) Oxygen consumption ($\dot{V}O_2$)) (**f**), 24 h $\dot{V}O_2$) (**g**), and locomotor activity (**h**) of male KI mice at 26 weeks of age. For *Sh* mice, WT males (black squares, n = 12), KI males (grey squares, n = 9), WT females (black circles, n = 12), and KI females (grey circles, n = 9) were analysed. Statistical analyses were performed using the two-tailed unpaired Student's *t* test (+, $p < 0.1$ KI vs WT; *, $p <$ 0.05 KI vs WT). Black bars and black lines, WT data; white bars and grey lines, KI data. M, male; F, female
