# Supplementary material for: Hypothalamic SIRT1 prevents age-associated weight gain by improving leptin sensitivity in mice
Source: Diabetologia. 2013 Dec 29;57(4):819–31. doi: 10.1007/s00125-013-3140-5 (PMC3940852; doi:10.1007/s00125-013-3140-5)
Supplement: Supplementary file 4 — (PDF 115 kb) [file 125_2013_3140_MOESM4_ESM.pdf]

ESM Fig. 3

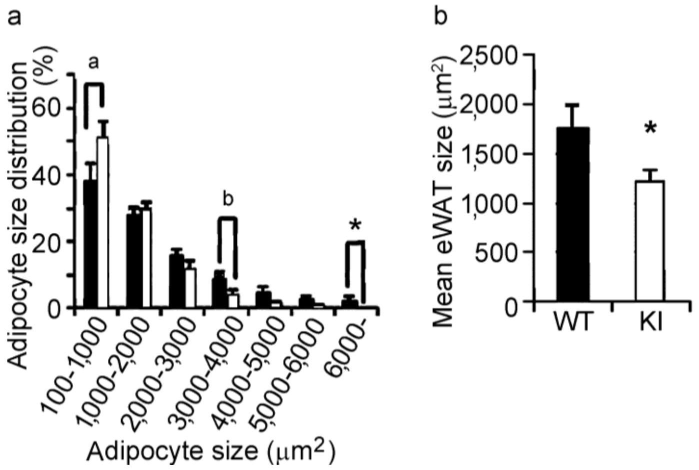

ESM Fig. 3, related to Fig. 2c-d. Phenotypes of *Pomc-Cre; Rosa26* $^{Sirt1\text{-}WT}$ mice. (**a**, **b**) Adipocyte size distribution (**a**), and mean adipocyte size (**b**) in the eWAT of male *Pomc-Cre; Rosa26* $^{Sirt1\text{-}WT}$ (*Sw*) mice at 26 weeks of age. Statistical analyses were performed using the two-tailed unpaired Student's *t* test (*, p<0.05 KI vs WT). There were trends in (**a**) (^{a}p=0.08; ^{b}p=0.07). Black bars, WT data; white bars, KI data
