# Supplementary material for: Hypothalamic SIRT1 prevents age-associated weight gain by improving leptin sensitivity in mice
Source: Diabetologia. 2013 Dec 29;57(4):819–31. doi: 10.1007/s00125-013-3140-5 (PMC3940852; doi:10.1007/s00125-013-3140-5)
Supplement: Supplementary file 5 — (PDF 136 kb) [file 125_2013_3140_MOESM5_ESM.pdf]

ESM Fig. 4

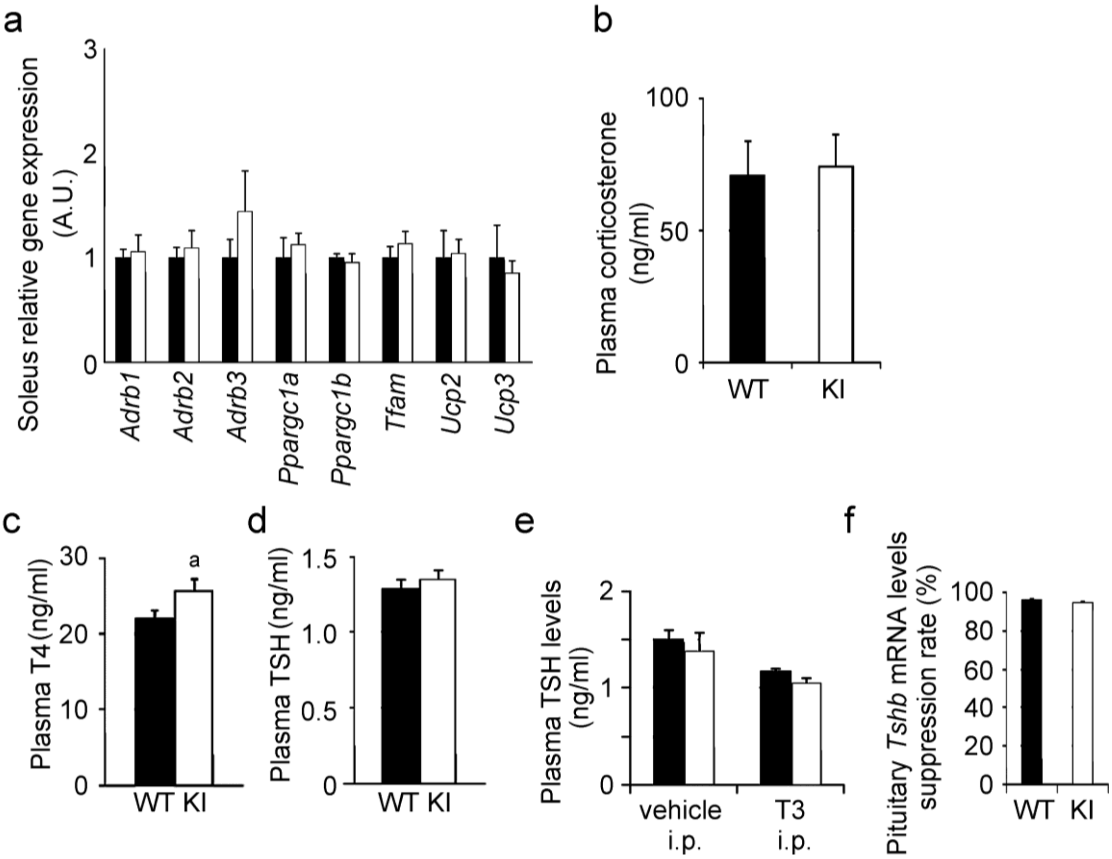

ESM Fig. 4, related to Fig. 3a-j. Phenotypes of *Pomc-Cre; Rosa26* $^{Sirt1\text{-}WT}$ mice. (**a**) Gene expression profiles in the soleus muscle of KI mice at 26 weeks of age. (**b-d**) Plasma corticosterone (**b**), T4 (**c**), and TSH (**d**) levels in KI mice at 26 weeks of age. (**e, f**) The effects of the T3 suppression test on plasma TSH levels (**e**) and pituitary *Tshb* mRNA levels (**f**) in 12-week-old male KI mice (WT, n = 4; KI, n = 7). The same number of mice was used as in Figure 3 unless otherwise indicated. Statistical analyses were performed using the two-tailed unpaired Student's *t* test. For (**c**), there was a trend ([a]p=0.08). Black bars, WT data; white bars, KI data
