# Supplementary material for: Hypothalamic SIRT1 prevents age-associated weight gain by improving leptin sensitivity in mice
Source: Diabetologia. 2013 Dec 29;57(4):819–31. doi: 10.1007/s00125-013-3140-5 (PMC3940852; doi:10.1007/s00125-013-3140-5)
Supplement: Supplementary file 6 — (PDF 123 kb) [file 125_2013_3140_MOESM6_ESM.pdf]

ESM Fig. 5

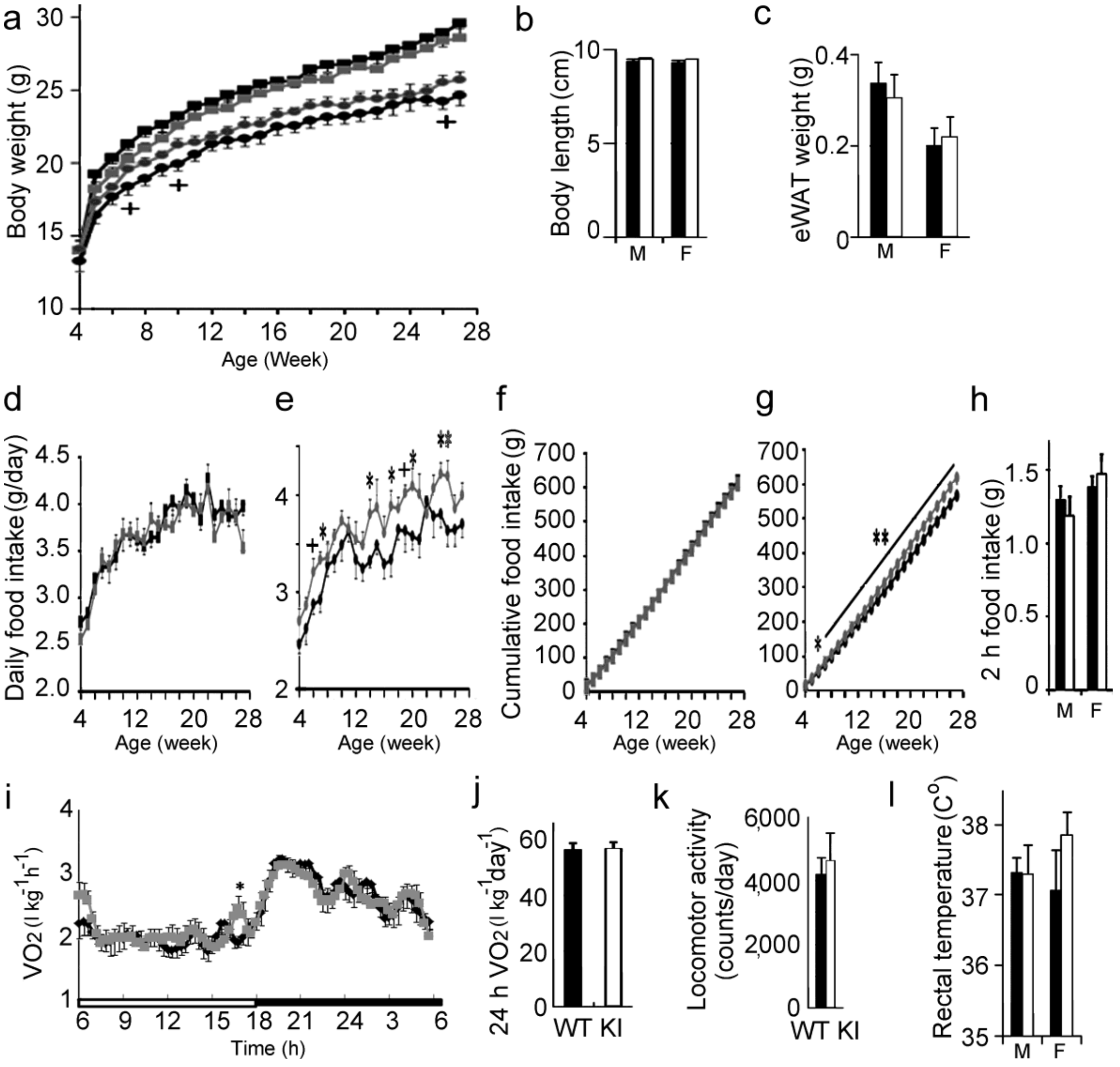

ESM Fig. 5, related to Fig. 4a-k. Phenotypes of *Agrp-Cre; Rosa26* $^{\mathit{Sirt1\text{-}H355Y}}$ mice. (**a**) Body-weight curves of *Agrp-Cre; Rosa26* $^{Sirt1\text{-}H355Y}$ (*Sh*) (black squares, WT males, n = 12; grey squares, KI males, n = 10; black circles, WT females n = 8; grey circles, KI females, n = 10) conditional KI mice. (**b, c**) Body length (**b**) and epididymal white adipose tissue (eWAT) weight (**c**) of KI mice at 28 weeks of age. (**d-h**) Daily food intake (male, **d**; female, **e**), cumulative food intake (male, **f**; female, **g**), and 2 h food intake (**h**) of KI mice after 24 h of fasting. (**i-k**) Oxygen consumption ($\dot{V}O_2$) (**i**), 24 h $\dot{V}O_2$ (**j**) and locomotor activity (**k**) of KI mice at 28 weeks of age. (**l**) Rectal temperature of KI mice at 28 weeks of age. The same number of mice was used as in Fig. 4 unless otherwise indicated. Statistical analyses were performed using the two-tailed unpaired Student's *t* test ($^{+}p<0.1$; $^{*}p<0.05$; $^{**}p<0.01$ KI vs WT). Black bars, WT data; white bars, KI data. M, male; F, female
