# Supplementary material for: Hypothalamic SIRT1 prevents age-associated weight gain by improving leptin sensitivity in mice
Source: Diabetologia. 2013 Dec 29;57(4):819–31. doi: 10.1007/s00125-013-3140-5 (PMC3940852; doi:10.1007/s00125-013-3140-5)
Supplement: Supplementary file 7 — (PDF 155 kb) [file 125_2013_3140_MOESM7_ESM.pdf]

ESM Fig. 6

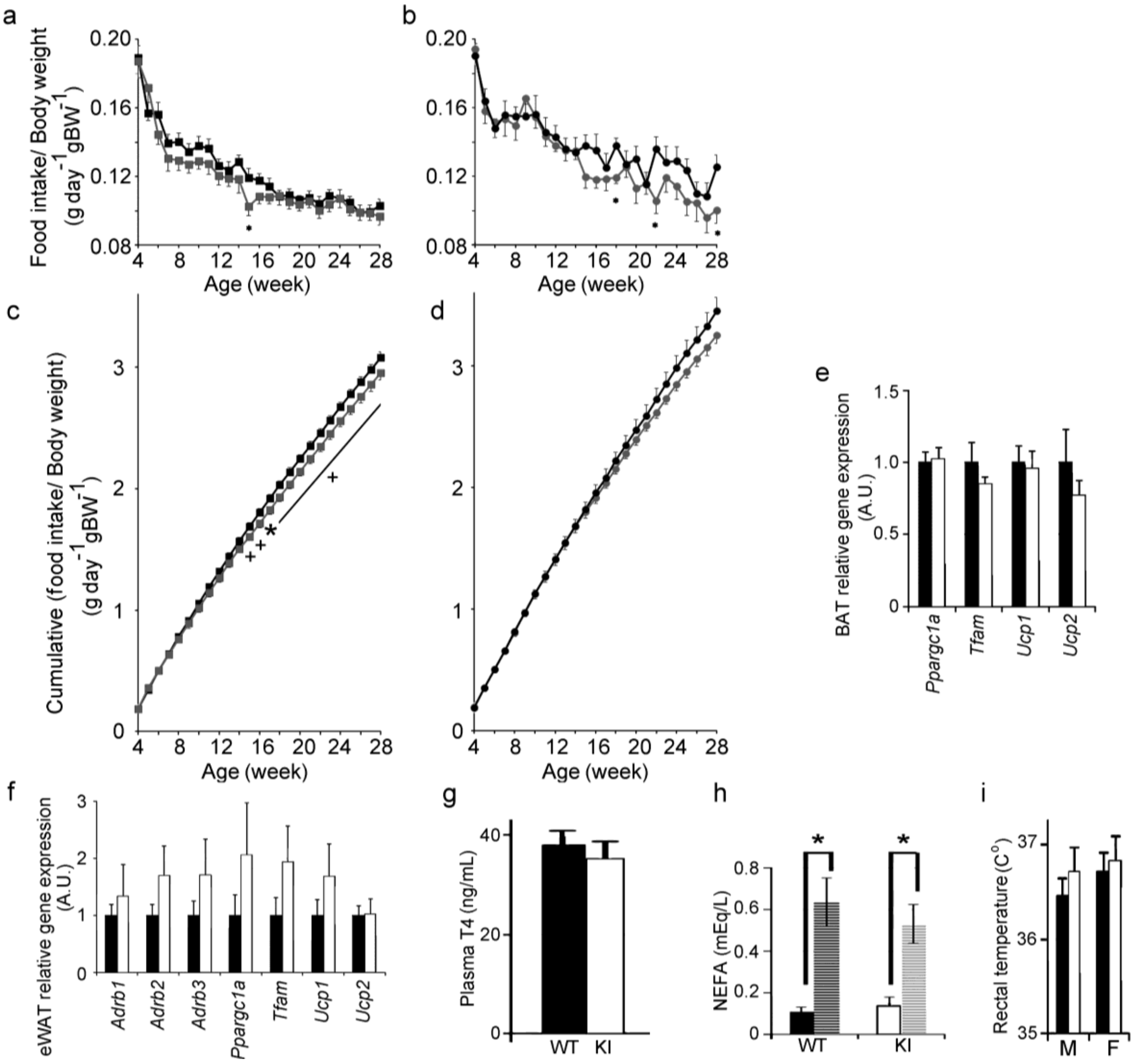

ESM Fig. 6, related to Fig. 4a-k. Overexpression of WT *Sirt1* in AgRP neurons prevents age-associated weight gain in male mice. (**a-d**) Food intake data corrected by body weight (male, **a**; female, **b**) and the cumulative change (male, **c**; female, **d**) of *Agrp-Cre; Rosa26* $^{Sirt1\text{-}WT}$ (*Sw*) (black squares, WT males, n = 18; grey squares, KI males, n = 12; black circles, WT females, n = 9; grey circles, KI females, n = 8) conditional KI mice. (**e, f**) Gene expression profiles of brown adipose tissue (**e**) and eWAT (**f**) of 28-week-old male KI mice. (**g**) Plasma T4 levels of 28-week-old male KI mice. (**h**) Plasma non-esterified fatty acid (NEFA) levels under fed and fasted conditions in KI mice (black, WT fed, n = 9; black stripe, WT fasted, n = 8; white, KI fed, n = 6; white stripe, KI fasted, n = 5). (**i**) Rectal temperature of KI mice. Statistical analyses were performed using the two-tailed unpaired Student's *t* test (*, $p < 0.05$; +, $p < 0.1$). Black bars, WT data; white bars, KI data. M, male; F, female
