# Supplementary material for: Hypothalamic SIRT1 prevents age-associated weight gain by improving leptin sensitivity in mice
Source: Diabetologia. 2013 Dec 29;57(4):819–31. doi: 10.1007/s00125-013-3140-5 (PMC3940852; doi:10.1007/s00125-013-3140-5)
Supplement: Supplementary file 8 — (PDF 147 kb) [file 125_2013_3140_MOESM8_ESM.pdf]

ESM Fig. 7

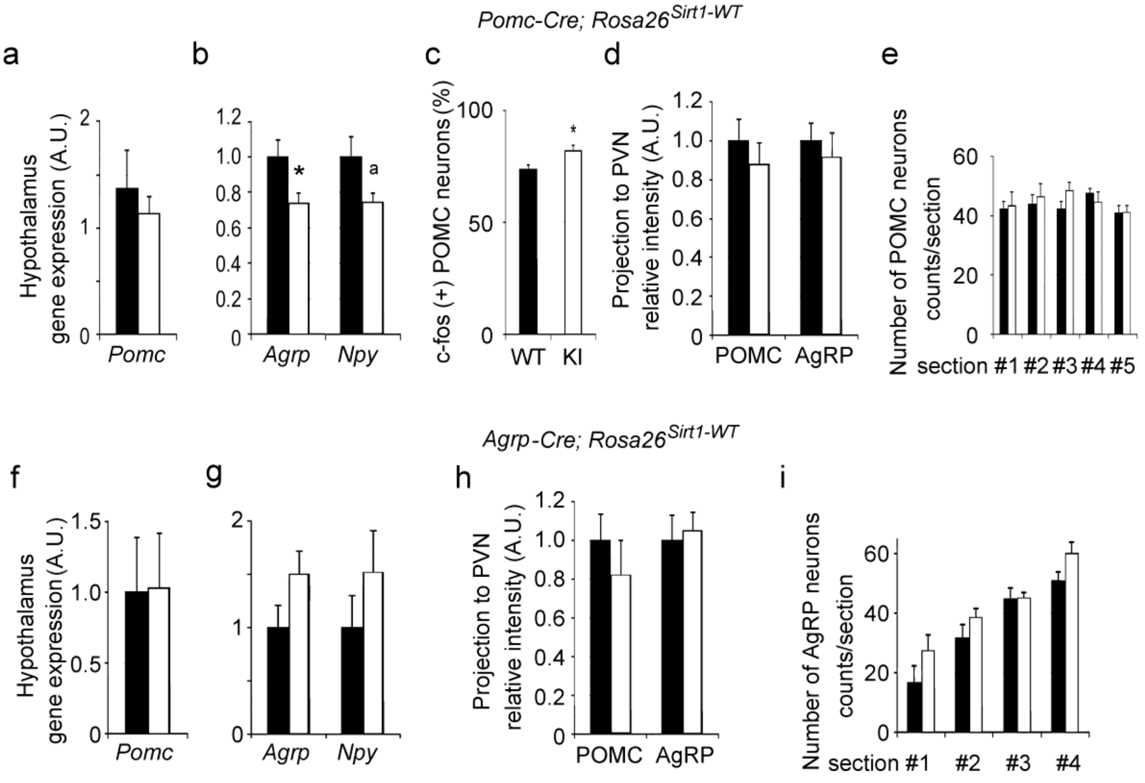

ESM Fig. 7, related to Fig. 5a-e. Gene expression analyses and immunohistological analyses of the hypothalamus of *Pomc-Cre; Rosa26* $^{Sirt1\text{-}WT}$ mice and *Agrp-Cre; Rosa26* $^{Sirt1\text{-}WT}$ mice. (**a, b**) Gene expression profiles in the hypothalamus of *Pomc-Sw* KI mice at 26 weeks of age. (**c**) The percentage of c-fos (+) POMC neurons after 24 h of fasting. (**d, e**) Immunohistological analyses of POMC and AgRP fibre projections to the paraventricular nucleus (PVN) (**d**) and the number of POMC neurons in ARC sections (**e**) in 12-week-old male *Pomc-Sw* KI mice (WT, n = 5; KI, n = 4). (**f, g**) Gene expression profiles of the hypothalamus of male *Agrp-Sw* KI mice at 28 weeks of age. (**h, i**) Immunohistological analysis of POMC and AgRP fibre projections to PVN (**h**) and the number of AgRP neurons in ARC sections (**i**) in *Agrp-Sw* KI mice (n = 4 or 5 per group). The same number of mice was used as in Fig. 3-4 unless otherwise indicated. Statistical analyses were performed using the two-tailed unpaired Student's *t* test (*, $p < 0.05$). For (**b**), there was a trend ($^{a}p = 0.07$). Black bars, WT data; white bars, KI data
