# Supplementary material for: Hypothalamic SIRT1 prevents age-associated weight gain by improving leptin sensitivity in mice
Source: Diabetologia. 2013 Dec 29;57(4):819–31. doi: 10.1007/s00125-013-3140-5 (PMC3940852; doi:10.1007/s00125-013-3140-5)
Supplement: Supplementary file 9 — (PDF 148 kb) [file 125_2013_3140_MOESM9_ESM.pdf]

ESM Fig. 8

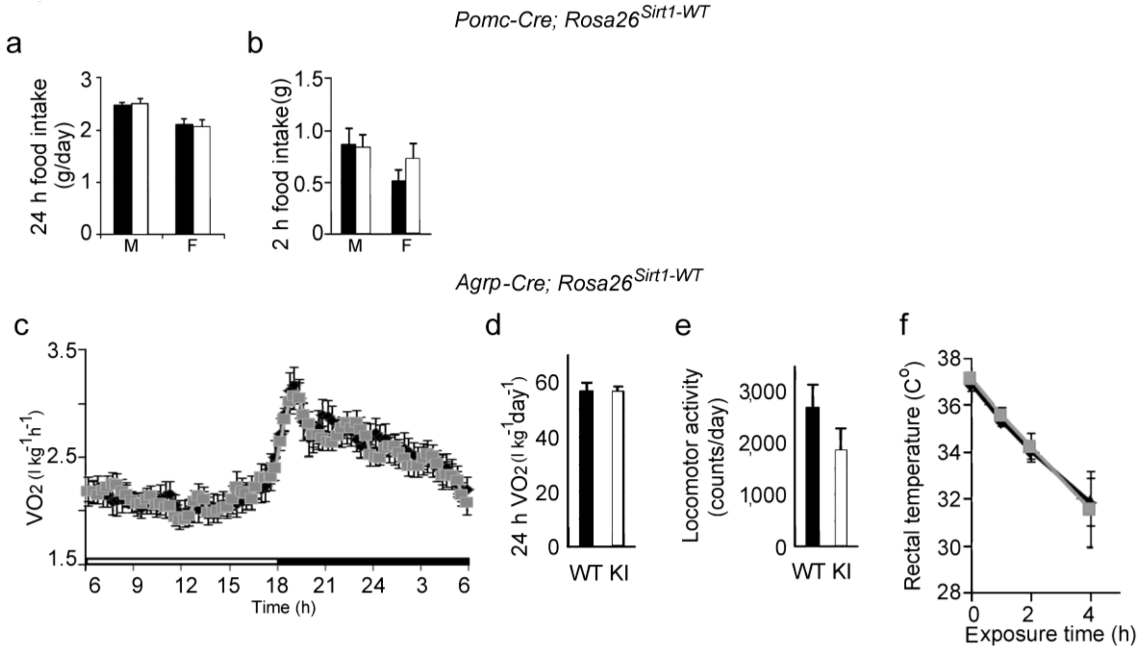

ESM Fig. 8, related to Fig. 6a-m. Phenotypes of *Pomc-Cre; Rosa26* $^{Sirt1\text{-}WT}$ mice and *Agrp-Cre; Rosa26* $^{Sirt1\text{-}WT}$ mice fed a HFHS diet. (**a, b**) 24 h food intake (**a**) and 2 h food intake after 24 h fasting (**b**) of male 26-week-old *Pomc-Sw* KI mice fed an HSHS diet. (**c-e**) Oxygen consumption ($\dot{V}O_2$) (**c**), 24 h $\dot{V}O_2$ (**d**) and locomotor activity (**e**) of HFHS diet-fed *Agrp-Sw* KI mice at 28 weeks of age. (**f**) Rectal temperature of male *Agrp-Sw* KI mice during a 4 h cold exposure (4 °C). The same number of mice was used as in Fig. 6 unless otherwise indicated. Statistical analyses were performed using the two-tailed unpaired Student’s *t* test. Black bars and black lines, WT data; white bars and grey lines, KI data. M, male; F, female
