# Supplementary material for: Hypothalamic SIRT1 prevents age-associated weight gain by improving leptin sensitivity in mice
Source: Diabetologia. 2013 Dec 29;57(4):819–31. doi: 10.1007/s00125-013-3140-5 (PMC3940852; doi:10.1007/s00125-013-3140-5)
Supplement: Supplementary file 10 — (PDF 26 kb) [file 125_2013_3140_MOESM10_ESM.pdf]

ESM Table 1

Sequences of the primers used for genotyping.

Genes	Primer sequence	Amplicon size (bp)
Cre sense	CTGCCACGACCAAGTGACAGC	300
Cre antisense	CTTCTCTACACCTGCGGTGCT	
Rosa26 genomic F	AAAGTCGCTCTGAGTTGTTAT	WT, 600; Rosa, 300
Rosa26 genomic R	GGAGCGGGAGAAATGGATATG	
Rosa26 SA R	GCGAAGAGTTTGTCCTCAACC	
SA end F	CAAACTCTTCGCGGTCTTTCC	600
Sirt1-394R	TCGTCGTCGTCGAAGTCGTCAGC	
Sirt1-1918F	GTAAGCGGCTTGAGGG	450
M13F	GTAAAACGACGGCCAGT	
M13R	CAGGAAACAGCTATGAC	
