# Supplementary material for: Hypothalamic SIRT1 prevents age-associated weight gain by improving leptin sensitivity in mice
Source: Diabetologia. 2013 Dec 29;57(4):819–31. doi: 10.1007/s00125-013-3140-5 (PMC3940852; doi:10.1007/s00125-013-3140-5)
Supplement: Supplementary file 11 — (PDF 29 kb) [file 125_2013_3140_MOESM11_ESM.pdf]

ESM Table 2

Sequences of the primers used for RT-PCR.

Gene	Gene ID	Forward primer sequence	Reverse primer sequence	Amplicon size (bp)
Actb	NM_007393	AGCCTTCCTTCTTGGGTA	GAGCAATGATCTTGATCTTC	207
Adrb1	NM_007419	CAGCATTGAGACCCTGTGTG	AGCACTTGGGGTCGTTGTAG	215
Adrb2	NM_007420	CGAGTGGTCATCCTGATG	GCAGAACTTGGAGGACC	345
Adrb3	NM_013462	TCCGTTTTTGCAGGACTTCT	ACGGTGAAACCCATTTGGTA	194
Agrp	NM_007427	GAGTTCCCAGGTCTAAGTCTGAATG	ATCTAGCACCTCCGCCAAAG	102
Cd137	NM_011612	CGTGCAGAACTCCTGTGATAAC	GTCCACCTATGCTGGAGAAGG	104
Cd36	NM_007643	TGGCCTTACTTGGGATTGG	CCAGTGTATATGTAGGCTCAT	211
Dio2	NM_010050	CCGCTCCAAGTCCACTCGCG	CGGCCCCATCAGCGGTCTTC	197
Leprb	NM_146146	GAGCCTGAACCCATTTCAGAAG	ACCATAGCTGCTGGGACCAT	80
Npy	NM_023456	TACTCCGCTCTGCGACACTA	TCTTCAAGCCTTGTTCTGGG	134
Pomc	NM_008895	GGCTTGCAAACTCGACCTCT	TGACCCATGACGTACTTCCG	99
Ppargc1a	NM_008904	AATTCTCCCTTGTATGTGAGA	TTACCTGCGCAAGCTTCTCTG	565
Ppargc1b	NM_133249	TGCCCCTACCAGAGCCCACC	CATGCCGGACGCTTGGGGTT	166
Prdm16	NM_027504	AAGCACGAACACGAGGGCGC	CCAGGTCTTGGATCTCAGGCCGTTT	220
Ptpn1	NM_011201	AGCCACCAGTGGGTGAGCGA	TCCTCGGTGGGGACAGACGC	179
Ptpn2	NM_001127177	AGGGCTTCCTTCTAAGGTGCAGGA	AAAAGCAGTGTCCAGCCAACCA	237
Sirt1	NM_019812	GTAAGCGGCTTGAGGG	TTCGGGCCTCTCCGTA	130
Socs3	NM_007707	TGTCGGAAGACTGTCAACGG	GAAGAAGCCAATCTGCCCCT	237
Tbx1	NM_011532	GGCAGGCAGACGAATGTTC	TTGTCATCTACGGGCACAAAG	102
Tfam	NM_009360	AAGACCTCGTTCAGCATATAACATT	TTTTCCAAGCCTCATTTACAAGC	104
Th	NM_009377	AGGTCCGGGCCTTTGACCCA	GCGCCGGATGGTGTGAGGAC	209
Tmem26	NM_177794	ACCCTGTCATCCCACAGAG	TGTTTGGTGGAGTCCTAAGGTC	120
Ucp1	NM_009463	TACAGAGTTATAGCCACCACA	CACAAACATGATGACGTTCCA	381
Ucp2	NM_011671	ACTGAGGGTCCACGCAGCCT	ATGCCTGCATGCTCTGAGCCC	134
Ucp3	NM_009464	TAAACAGGTGAGACTCCAGCAACTT	ACTCCAGCGTCGCCATCAGGATTCT	300
