# Supplementary material for: Hypothalamic SIRT1 prevents age-associated weight gain by improving leptin sensitivity in mice
Source: Diabetologia. 2013 Dec 29;57(4):819–31. doi: 10.1007/s00125-013-3140-5 (PMC3940852; doi:10.1007/s00125-013-3140-5)
Supplement: Supplementary file 12 — (PDF 42 kb) [file 125_2013_3140_MOESM12_ESM.pdf]

ESM Table 3

Antibodies used for histological studies:	
AgRP	Santa Cruz Biotechnology, sc-18634
c-fos	Santa Cruz Biotechnology, sc-52 or sc-52G
Phospho-STAT3	Cell Signaling Technology, #9131s
POMC	Phoenix Pharmaceuticals, H-029-30
	Santa Cruz Biotechnology, sc-18264
Sirt1	abcam, ab50517
UCP1	abcam, ab10983
**Antibodies used for western analyses:**	
α-tubulin	Santa Cruz Biotechnology, sc-5286
Acetylated FKHR	Santa Cruz Biotechnology, sc-49437-R
DYKDDDDK (FLAG)	Wako Pure Chemical Industries, 018-22381
FoxO1	Cell Signaling Technology, #9462
PTP1B	Santa Cruz Biotechnology, sc-1718
Phospho-Tyr705-STAT3	Cell Signaling Technology, #9131
Sirt1	Millipore, #07-131
SOCS3	Cell Signaling Technology, #2923
STAT3	Cell Signaling Technology, #9362
TC-PTP	Santa Cruz Biotechnology, sc-21345-R

Antibody distributors:

abcam (Tokyo, Japan)

Cell Signaling Technology (Tokyo, Japan)

Millipore (Billerica, MA, USA)

Phoenix Pharmaceuticals (Burlingame, CA, USA)

Santa Cruz Biotechnology (Dallas, Texas, USA)

Wako Pure Chemical Industries (Osaka, Japan)
